# Comparative Analysis of Transcriptomic Response of Escherichia coli K-12 MG1655 to Nine Representative Classes of Antibiotics

**DOI:** 10.1128/spectrum.00317-23

**Published:** 2023-02-28

**Authors:** Luyao Bie, Mengge Zhang, Juan Wang, Meng Fang, Ling Li, Hai Xu, Mingyu Wang

**Affiliations:** a State Key Laboratory of Microbial Technology, Microbial Technology Research Institute, Shandong University, Qingdao, China; b Tsinghua University-Peking University Joint Center for Life Sciences, School of Life Sciences, Tsinghua University, Beijing, China; c No.3 Middle School of Huimin, Binzhou, China; Department of Clinical Laboratory, Peking University People’s Hospital, Beijing, China

**Keywords:** *Escherichia coli*, antibiotics, transcriptomics, stress response, environment adaptation, comparative analysis

## Abstract

The use of antibiotics leads to strong stresses to bacteria, leading to profound impact on cellular physiology. Elucidating how bacteria respond to antibiotic stresses not only helps us to decipher bacteria’s strategies to resistant antibiotics but also assists in proposing targets for antibiotic development. In this work, a comprehensive comparative transcriptomic analysis on how Escherichia coli responds to nine representative classes of antibiotics (tetracycline, mitomycin C, imipenem, ceftazidime, kanamycin, ciprofloxacin, polymyxin E, erythromycin, and chloramphenicol) was performed, aimed at determining and comparing the responses of this model organism to antibiotics at the transcriptional level. On average, 39.71% of genes were differentially regulated by antibiotics at concentrations that inhibit 50% growth. Kanamycin leads to the strongest transcriptomic response (76.4% of genes regulated), whereas polymyxin E led to minimal transcriptomic response (4.7% of genes regulated). Further GO, KEGG, and EcoCyc enrichment analysis found significant transcriptomic changes in carbon metabolism, amino acid metabolism, nutrient assimilation, transport, stress response, nucleotide metabolism, protein biosynthesis, cell wall biosynthesis, energy conservation, mobility, and cell-environmental communications. Analysis of coregulated genes led to the finding of significant reduction of sulfur metabolism by all antibiotics, and analysis of transcription factor-coding genes suggested clustered regulatory patterns implying coregulation. In-depth analysis of regulated pathways revealed shared and unique strategies of E. coli resisting antibiotics, leading to the proposal of four different strategies (the pessimistic, the ignorant, the defensive, and the invasive). In conclusion, this work provides a comprehensive analysis of E. coli’s transcriptomic response to antibiotics, which paves the road for further physiological investigation.

**IMPORTANCE** Antibiotics are among the most important inventions in the history of humankind. They are the ultimate reason why bacterial infections are no longer the number one threat to people’s lives. However, the wide application of antibiotics in the last half a century has led to aggravating antibiotic resistance, weakening the efficacy of antibiotics. To better comprehend the ways bacteria deal with antibiotics that may eventually turn into resistance mechanisms, and to identify good targets for potential antibiotics, knowledge on how bacteria regulate their physiology in response to different classes of antibiotics is needed. This work aimed to fill this knowledge gap by identifying changes of bacterial functions at the transcription level and suggesting strategies of bacteria to resist antibiotics.

## INTRODUCTION

Stress response pathways are key tools for bacteria to adapt to fluctuating environments. They allow cells to react to unfavored or even lethal environmental conditions, survive harsh times, and preserve cellular functional integrity after damage. These stress response pathways cover a wide range of cellular physiological aspects, including cell cycle regulation, chromosome maintenance, DNA damage repair, induction of molecular chaperones, degradation of damaged proteins, and certain aspects of metabolism (1, 2). The stress response to various signals has been a central topic of microbiology in the past century.

Antibiotics are an ancient yet also new environmental stress signal that modern bacteria encounter. These bactericidal chemical agents were originally synthesized by a variety of organisms, such as multicellular fungi and soil bacteria, and existed on Earth for millions of years (3). On the other hand, the discovery of antibiotics in the early 1900s and their subsequent wide medicinal applications have led to a burst of synthesis and usage of both old and novel, previously unseen antibiotics, resulting in significantly elevated antibiotic levels in the environment and a much higher frequency of bacteria meeting with high concentrations of antibiotics (4). Therefore, although stress response mechanisms against antibiotics should have existed for a very long time, they have met unprecedented stress for evolution in the past century, which is a new issue to solve.

To date, several dozens of classes of antibiotics have been used in medical and agricultural sectors. As different classes of antibiotics have distinct primary cellular targets, the bacterial killing mechanism is specific to each antibiotic class. For instance, β-lactams induce a lethal malfunctioning of the bacterial cell wall synthesis (5), aminoglycosides lead to mistranslation of peptides and membrane damage (6), fluoroquinolones cause DNA cleavage by inhibiting DNA gyrase or topoisomerase IV (7), and tetracyclines inhibit protein synthesis by binding to the 30S ribosomal subunit (8). Therefore, the types of stress that bacteria are exposed to by different classes of antibiotics are by nature different, although similarities exist as universal mechanisms to prevent cellular damage is present.

Determining these similarities and differences in how bacteria respond to antibiotics is of interest both scientifically and practically. The biggest threat that jeopardizes antibiotic application today is antimicrobial resistance (AMR). AMR is a mechanism that reduces or even eliminates the bactericidal effects of antibiotics. It is projected to become the biggest cause of death by 2050, leading to 10 million lost lives annually worldwide if the current AMR situation continues (9). AMR emerges and develops in response to the evolutionary pressure of antibiotics. Therefore, bacterial stress responses to antibiotics not only are a mechanism for bacteria to resist antibiotics but can also lead to the development of new AMR mechanisms. For these reasons, a comprehensive analysis of bacterial response to antibiotics is required for understanding both the mechanism which bacteria exploit to reduce antibiotic effectiveness and how new AMR mechanisms may evolve.

Transcriptomic and proteomic responses of bacteria toward some antibiotics have been reported. Exposure of Escherichia coli to β-lactams, rifampin, aminoglycosides, fluoroquinolones, tetracycline (TET), and imipenem (IPM) leads to the induction of the RpoS regulon (10), increased expression of *rpoB* and several genes involved in nucleotide salvage and purine biosynthesis (10), induction of heat shock proteins (11), increased transcription of genes participating in the SOS pathway (12, 13), promotion of glycolysis/gluconeogenesis metabolism (12), and induction of the oxidative stress response (14). A recent study also compared the transcriptomes of different E. coli strains, including E. coli K-12 MG1655, investigated in this work (15). For Haemophilus influenzae, chloramphenicol (CHL), erythromycin (ERY), fusidate, and tetracycline lead to increase of total RNA synthesis under the control of the stringent factor guanosine 5′,3′-bis-diphosphate (ppGpp), while rifampin leads to fast cessation of RNA synthesis (16); novobiocin and ciprofloxacin (CIP) induce DNA gyrase and represses topoisomerase I expression, while ciprofloxacin also induces the SOS response (17). For Pseudomonas aeruginosa, tobramycin leads to heat shock response and stimulated efflux mechanisms (18). For Acinetobacter baumannii, amikacin, imipenem, meropenem, or tigecycline treatment leads to increased expression of genes associated with transposable elements (19, 20), ceftazidime treatment leads to increased expression of toxin/antitoxin systems (21), and polymyxin leads to upregulation of membrane biogenesis and homeostasis, lipoprotein and phospholipid trafficking, and efflux pump and poly-*N*-acetylglucosamine biosynthesis, as well as downregulation of fatty acid biosynthesis (22). In addition to Gram-negative bacteria, transcriptional responses of Gram-positive bacteria to bactericidal agents have also been reported, including the response of Bacillus subtilis to chloramphenicol, erythromycin, gentamicin (23); the response of Streptomyces coelicolor to vancomycin, moenomycin A, and bacitracin (24); the response of Streptococcus pneumoniae to puromycin, tetracycline, chloramphenicol, and erythromycin (25); the response of Staphylococcus aureus to oxacillin, d-cycloserine, bacitracin, and ampicillin (26, 27); and the response of Mycobacterium tuberculosis to isoniazid (28).

Despite this progress, systematic investigations and comparisons of bacterial responses to antibiotics are still needed. Novel approaches were used in recent years to predict mode of action by transcriptomic or proteomic profiling of different antibiotics as performed in E. coli (29, 30), but these investigations were primarily aimed at developing antibiotic mechanism prediction models. Therefore, in this work, aimed at a systematic analysis of transcriptomic response of bacteria to different classes of antibiotics and elucidating shared and unique responses of different antibiotics, we used Escherichia coli K-12 strain MG1655 as a model system to analyze the dynamic transcriptional response to subinhibitory concentration of nine different representative classes of antibiotics—tetracycline, mitomycin C (MMC), imipenem, ceftazidime (CAZ), kanamycin (KAN), ciprofloxacin, polymyxin E (PME), erythromycin, and chloramphenicol—providing a comprehensive interpretation and comparison of the responses of E. coli to various classes of antibiotics.

## RESULTS

### Transcriptomes of E. coli exposed to subinhibitory concentrations of antibiotics.

In order to get a comprehensive picture of how E. coli responds to the pressure of antibiotics, we performed a transcriptomic analysis of E. coli treated with nine different antibiotics, each representing a major class of antibiotics: tetracycline (TET; tetracyclines), mitomycin C (MMC; mitomycins), imipenem (IPM; carbapenems), ceftazidime (CAZ; β-lactams), kanamycin (KAN; aminoglycosides), ciprofloxacin (CIP; quinolones), polymyxin E (PME; colistins), erythromycin (ERY; macrolides), and chloramphenicol (CHL; amphenicols). Most of these drugs have clinical applications. The responses of E. coli growth to treatment with different concentrations of antibiotics were determined (see Fig. S1 in the supplemental material). The antibiotic concentrations that inhibited 50% of growth (IC_50_) were used for transcriptomic analysis (Table S1).

Treatment with antibiotics led to dramatic changes in the transcriptomes of E. coli (Table 1 and Table S2). An average of 1,786 genes were differentially expressed under antibiotic treatment using an adjusted *P* value of <0.05 (with the Benjamini-Hochberg procedure) as a threshold, accounting for 39.7% of the genome. The average numbers of genes upregulated and downregulated under antibiotic stress were roughly equal (887 versus 899). Different antibiotics led to drastically different responses to the transcriptomes of E. coli. For kanamycin-treated E. coli, which was affected the greatest, 76.4% of the genome was differentially expressed, while for polymyxin E-treated E. coli, which was affected the least, only 4.7% of the genome was differentially expressed.

**TABLE 1:** Properties of antibiotic-treated and control transcriptomes^*a*^

Antibiotic	No. of combined clean reads	No. of upregulated genes	No. of downregulated genes	No. of differentially expressed genes (% of genome)
TET	47,210,378	1,084	1,134	2,218 (49.3)
MMC	46,660,344	840	891	1,731 (38.5)
IPM	47,220,294	589	703	1,292 (28.7)
CAZ	45,569,046	654	719	1,373 (30.5)
KAN	41,109,090	1,821	1,614	3,435 (76.5)
CIP	41,592,902	1,177	1,135	2,312 (51.4)
PME	44,994,738	78	135	213 (4.7)
ERY	43,815,626	1,010	1,000	2,010 (44.7)
CHL	55,024,204	734	760	1,494 (33.2)
Water	44,581,540	NA	NA	NA
ETH	56,837,348	NA	NA	NA

a Three replicates were performed in all cases. NA, not applicable; ETH, ethanol.

**FIG 1:**
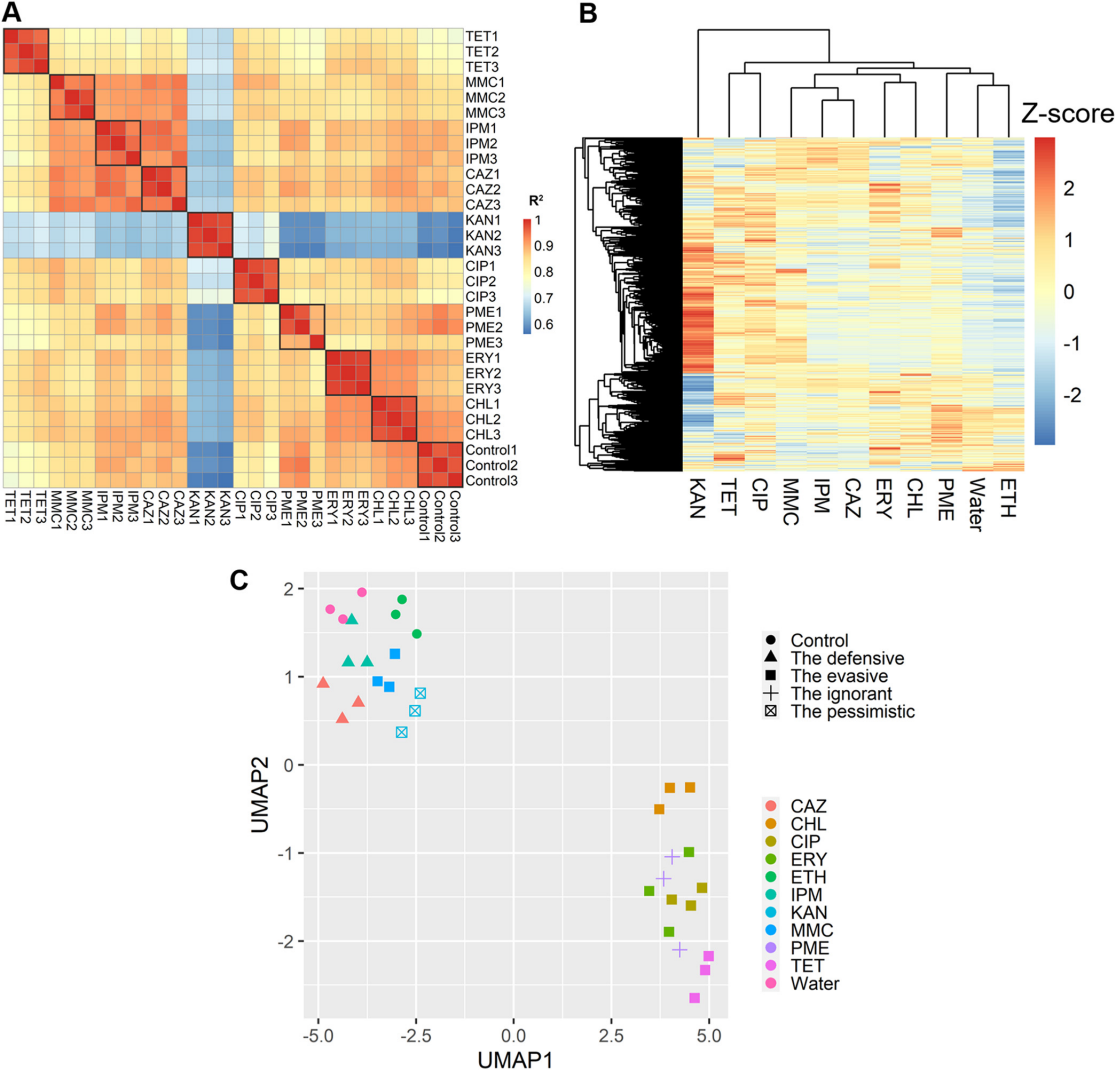
Comparison of transcriptomes of antibiotic-treated organisms. (A) Pearson correlation analysis of transcriptomes; (B) hierarchal clustering of transcriptomes; (C) UMAP analysis of transcriptomes.

By calculating and plotting the Pearson correlation between each transcriptome analyzed (Fig. 1A), we were able to see a good correlation between replicates (shown in black squares in Fig. 1A), confirming the validity of the methods undertaken. One observation is that the correlations between transcriptomes of kanamycin-treated organisms and other transcriptomes are particularly low (Fig. 1A), suggesting that kanamycin led to a change of transcriptomes drastically different from the case with other antibiotics. This is further supported by clustering analysis of the gene expression profiles following treatment with each chemical (Fig. 1B).

**FIG 2:**
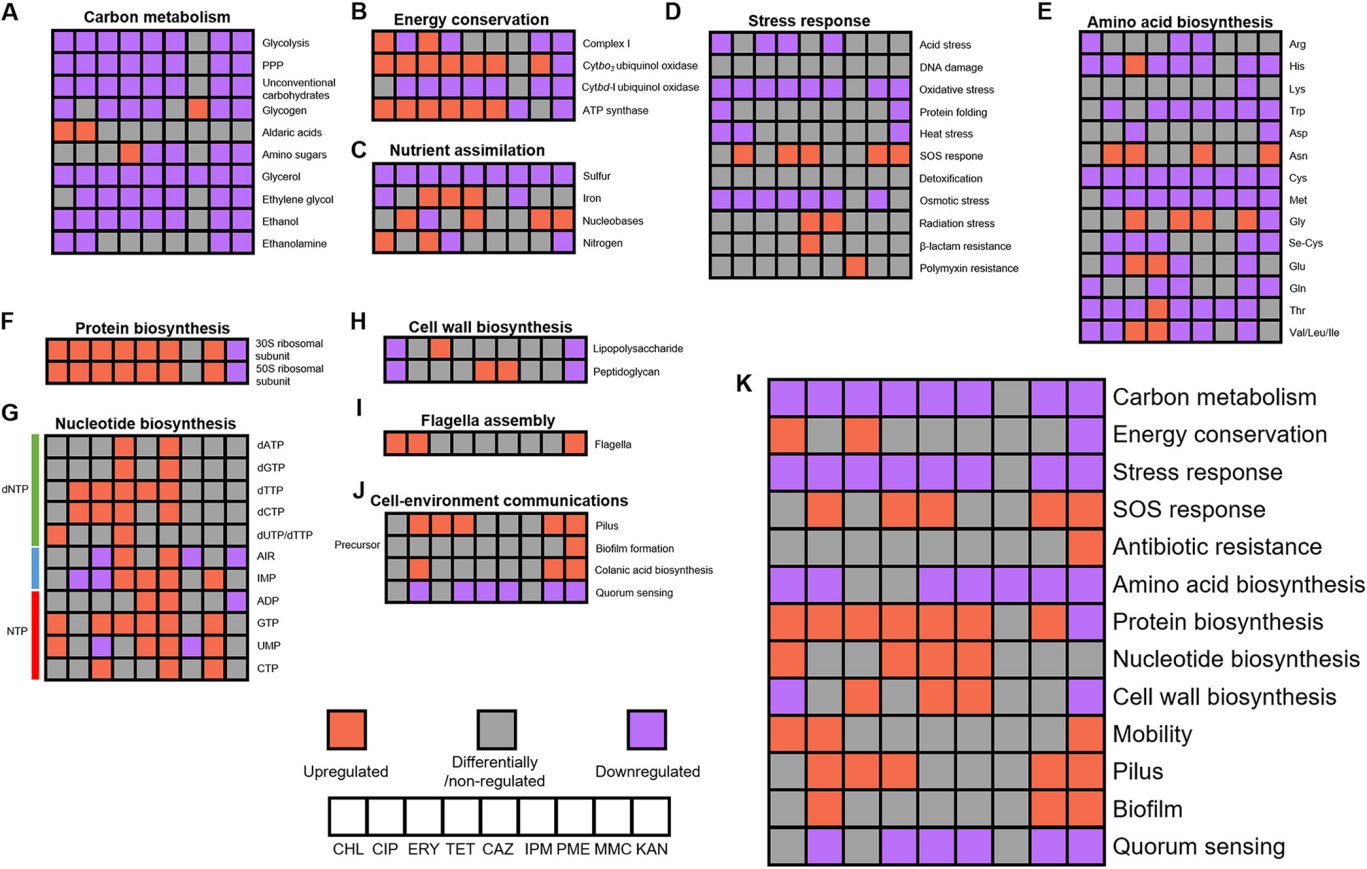
Transcriptomic response of metabolic pathways to antibiotics. (A) Carbon metabolism; (B) amino acid biosynthesis; (C) nutrient assimilation; (D) stress response; (E) nucleotide biosynthesis; (F) protein biosynthesis; (G) nucleotide biosynthesis; (H) cell wall biosynthesis; (I) flagellar assembly; (J) cell-environment communications; (K) comparison of physiological aspects impacted by antibiotics. AIR, 5-aminoimidazole ribonucleotide.

Further functional enrichment analysis with gene ontology (GO) (Fig. S2 to S19), KEGG (Table S3), and EcoCyc (Table S4) identified significant transcriptomic changes in carbon metabolism, amino acid metabolism, nutrient assimilation, transport, stress response, nucleotide metabolism, protein biosynthesis, cell wall biosynthesis, energy conservation, mobility, cell-environmental communications, and other cellular functions, which are further reported in this section (Fig. 2).

### Transcriptomic responses in carbon metabolism and energy conservation.

Carbon (particularly carbohydrate) metabolism and energy conservation lie at the very core of cellular function. Analysis of the transcriptomic response of carbon metabolism pathways revealed a generally downregulating pattern, with the exception of PME (Fig. 2A). For carbohydrate metabolism, the central glycolysis pathway was downregulated by all antibiotics but PME (Fig. S20A). For another key carbohydrate metabolism pathway, the pentose phosphate pathway (PPP), at least one of the three key genes *zwf*, *pgl*, and *gnd* in the oxidative branch was downregulated upon treatment with antibiotics except for PME (Fig. S20B). Although divergent responses were found for the nonoxidative branch of PPP, *talA* and *tktB*, which encode transaldolase and transketolase, the two most important enzymes, were downregulated on all antibiotics but PME (Fig. S20C). Metabolic pathways for unconventional carbohydrates, including rhamnose, trehalose, mannose, and xylose, were also similarly regulated (Fig. S20D to H). Combined, these data support a downregulation of carbohydrate metabolism pathways upon treatment with antibiotics except for PME.

Polysaccharides, more specifically glycogen, can serve as energy reserves for E. coli. The metabolism of this carbohydrate was transcriptionally regulated similarly with other carbohydrate metabolism pathways upon treatment with antibiotics (Fig. S21). Downregulation of the glycogen metabolism pathway can be observed with CHL, ERY, TET, CAZ, MMC, and KAN, although *pgm*, which encodes a phosphoglucomutase catalyzing conversion of α-d-glucopyranose 1-phosphate to d-glucopyranose 6-phosphate, was upregulated by CHL and ERY. Unlike other antibiotics, PME led to upregulation of this pathway.

Carbon sources other than carbohydrates can also be utilized by E. coli, and regulation of their metabolic pathways was also observed in response to antibiotic treatment (Fig. S22). Upregulation of metabolism for aldaric acids galactarate and glucarate can be found for CHL and CIP (Fig. S22A). Metabolic pathways of the carboxylates glycolate and glyoxylate were upregulated on CIP and downregulated on IPM, MMC, and KAN (Fig. S22B). Metabolism pathways of amino sugars were differentially regulated by different antibiotics: they were upregulated by TET and downregulated by CAZ, IPM, MMC, and KAN (Fig. S22C). For alcohols, glycerol metabolism pathways were downregulated on all antibiotics (Fig. S22D), ethylene glycol metabolism was downregulated on all antibiotics but CHL and PME (Fig. S22E), and ethanol metabolism was downregulated on all antibiotics but PME if we do not consider EutE as a major aldehyde dehydrogenase in ethanol metabolism (Fig. S22F). The ethanolamine metabolism pathway was downregulated in response to CHL, CIP, MMC, and KAN (Fig. S22G).

Analysis of transcriptomic response in energy conservation focused on four key complexes, NADH:quinone oxidoreductase I (complex I), cytochrome *bo_3_* ubiquinol oxidase, cytochrome *bd-I* ubiquinol oxidase, and the ATP synthase. The first three complexes are central for respiration, and the fourth complex is the primary machinery for ATP synthesis (Fig. 2B and Fig. S23). The two ubiquinol oxidases showed opposite regulatory patterns on nearly all antibiotics, except for KAN. Consistent regulation in energy conservation was found for CHL and KAN: upregulation for CHL and downregulation for KAN (Fig. S23). The most important respiratory complex, complex I, was also downregulated for CIP, TET, and MMC and upregulated on ERY (Fig. S23). ATP biosynthesis was upregulated on CIP, ERY, TET, CAZ, and IPM and downregulated for PME (Fig. S23).

### Transcriptomic responses on nutrient assimilation.

Significant transcriptomic responses were observed on nutrient assimilation pathways under the stress of antibiotics (Fig. 2C). Sulfur assimilation by reducing sulfate to hydrogen sulfide was downregulated under the stress of all antibiotics (Fig. S24A), consistent with the downregulation of ABC transporter-coding genes for sulfate and thiosulfate (Fig. S25), suggesting global downregulation of sulfur assimilation. Iron assimilation represented by siderophore biosynthesis was downregulated by CHL and PME but upregulated by ERY, TET, and CAZ (Fig. S24B). The downregulation of iron uptake on CHL and PME and upregulation on ERY were also confirmed by analysis of transporter-coding genes (Fig. S25). Transport of nucleobases (GO 0015292, nucleobase transmembrane transport activity) was upregulated on CIP, CAZ, MMC, and KAN and downregulated on ERY (Fig. S24C). The central nitrogen assimilation pathways, including nitrate, nitrite, and ammonia assimilation, were upregulated on CHL and ERY and downregulated on TET and KAN (Fig. S24D and S26). For cyanate metabolism, the conversion between carbamate and cyanate was upregulated on CHL and ERY and downregulated when exposed to all other antibiotics, whereas the phosphorylation of carbamate was upregulated on KAN (Fig. S24D and S26).

Uptake of nutrient was further analyzed by investigation of genes mapped to KEGG pathway ABC transporters (eco02010) and phosphotransferase system (PTS) transporters (eco02060), as well as genes mapped to transporter activity gene ontology term (GO 0005215) (Fig. S25). Responses to antibiotics appear to vary with each antibiotic. For nitrogen sources, uptake of sulfate/thiosulfate and putrescine was found to be downregulated for almost all antibiotics. Uptake of peptides, defensin, glutathione, and microcin C was downregulated after antibiotic treatment. Among metal ions, molybdate uptake was upregulated in response to all antibiotics except for TET and PME. Significant downregulation of uptake of carbohydrates could be found upon treatment with CAZ and IPM, whereas little response could be observed after PME treatment. This is also in agreement with the downregulation of carbon metabolism upon treatment of CAZ and IPM (Fig. 2A).

### Transcriptomic response in stress response.

Stress response pathways were differentially regulated with different stresses (Fig. 2D and Fig. S27 and S28). A global downregulation of oxidative stress response (GO 0006979, response to oxidative stress) and osmotic stress response (GO 0006970, response to osmotic stress) could be observed upon treatment with antibiotics other than PME. This is in contrast to the general upregulation of uptake of glycine betaine that is involved in osmotic stress response following treatment with CHL, CIP, ERY, TET, and KAN (Fig. S25). Responses to acid stress (GO 0010447, response to acidic pH), heat stress (GO 0009408, response to heat), and protein folding (GO 0006457, protein folding), on the other hand, were downregulated with only a few antibiotics. The downregulation of these pathways suggests different strategies of E. coli to deal with different stresses.

Upregulation of the SOS response pathway (GO 0009432, SOS response) could be observed with the treatment of CIP, TET, CAZ, MMC, and KAN (Fig. 2D), suggesting the key role of this pathway in response to external stress. Interestingly, radiation stress response (GO 0009314, response to radiation) was upregulated after treatment with cell wall inhibitors CAZ and IPM (Fig. 2D).

Another key aspect of stress response is the response to antibiotics and heavy metals. The β-lactam resistance pathway (KEGG eco01501) was upregulated following treatment with the β-lactam CAZ, and the polymyxin resistance pathway (EcoCyc PWY0-1338) was upregulated following treatment with PME (Fig. 2D and Fig. S27B and S28), suggesting that the regulation of these pathways is specific. For antibiotic resistance-conferring efflux pumps, the majority of the pumps were upregulated after KAN treatment, suggesting an increase of antibiotic resistance (Fig. S25). Antibiotic resistance-related outer membrane protein-coding genes, *ompA*, *ompC*, and *ompN*, were downregulated upon antibiotic treatment except for PME, suggesting that antibiotic resistance via downregulation of outer membrane proteins is an essential aspect of antibiotic stress response (Fig. S25). For metal ion efflux pumps that function in heavy metal resistance, a general downregulation pattern was found for copper tolerance genes *copA*, *ybaT*, Zn^2+^/Cd^2+^/Pb^2+^-transporting *zntA*, and arsenic-transporting *arsB*, whereas copper/silver-exporting *cusABC*, manganese-exporting *mntP*, and nickel/cobalt-exporting *rcnA* were generally upregulated (Fig. S25). The different regulatory patterns of heavy metal resistance genes and antibiotic resistance genes suggest that antibiotic resistance response is specific under antibiotic stress.

### Transcriptomic response in biosynthesis.

Biosynthesis of key cellular molecules and components was shown to be regulated by antibiotics in primarily four aspects: amino acid biosynthesis, protein biosynthesis, nucleotide biosynthesis, and cell wall biosynthesis. Functional enrichment analysis suggested significant changes in amino acid biosynthesis pathways following treatment with nearly all antibiotics. Amino acid biosynthesis was in general downregulated after antibiotic treatment (Fig. 2E), with several exceptions. Clear regulatory patterns could be observed on the metabolism/biosynthesis of 16 out of the 21 common amino acids (selenocysteine included). From the perspective of amino acids, metabolic pathways for histidine (Fig. S29C), tryptophan (Fig. S29E), cysteine (Fig. S29G), methionine (Fig. S29H), and threonine (Fig. S29K) were downregulated with the stress of nearly any investigated antibiotic. This is in agreement with downregulated transporter-coding genes for arginine, cysteine, and methionine (Fig. S25). Metabolic pathways for asparagine (Fig. S29F) and glycine (Fig. S29L) showed an upregulation pattern following antibiotic treatment. From the perspective of antibiotics, regulation of amino acid metabolic pathways was observed for every antibiotic. A general downregulation was found except for ERY and TET, which showed upregulation of metabolic pathways for several amino acids, such as glutamine (Fig. S29J), valine, leucine, and isoleucine (Fig. S29A). This downregulatory pattern was consistent with downregulation of amino acid uptake genes after treatment with CAZ, IPM, PME, and MMC (Fig. S25). Relatively weaker regulation was found for lysine metabolism (Fig. S29D) and for PME (Fig. 2E).

Regulation of protein biosynthesis (ribosomal protein-coding genes) showed a strong coregulation pattern (Fig. S30). The ribosomal protein-coding genes were upregulated following treatment with all antibiotics except for PME and KAN (Fig. 2F). No changes were observed for PME, and a downregulation of ribosomal protein-coding genes was observed for KAN (Fig. 2F).

The nucleotide biosynthesis pathways were significantly regulated by antibiotics (Fig. 2G and Fig. S31). In general, CHL, TET, CAZ, and IPM treatment led to global upregulation of the nucleotide biosynthesis pathway. Treatment with PME, MMC, and KAN led to consistent transcriptomic responses restricted to the biosynthesis of nucleoside triphosphates (NTPs) and their precursors: PME and KAN led to downregulation, while MMC led to upregulation. Varied responses in the biosynthesis of deoxynucleoside triphosphate (dNTP), NTP, and NTP precursors were found for CIP and ERY: treatment with CIP and ERY led to upregulation of dNTP biosynthesis pathways and downregulation of NTP precursor biosynthesis pathways. Meanwhile, treatment with ERY led to upregulation of GTP and CTP biosynthesis and downregulation of UMP biosynthesis.

Cell wall biosynthesis was general differentially regulated on different antibiotics (Fig. S32). Lipopolysaccharide biosynthesis was downregulated on CHL and KAN, whereas it was upregulated on ERY (Fig. 2H). For the biosynthesis of peptidoglycan, downregulation could also be observed on CHL and KAN, while cell wall inhibitors CAZ and IPM led to upregulation of these genes, whose products include their targets (Fig. 2H).

### Transcriptomic response in cell movement and communication.

Cell movement and communication are common approaches for bacteria to respond to stress. Regulation of flagellar assembly was found to be upregulated on three antibiotics CHL, CIP, and KAN. Genes encoding the basal body and rod were upregulated on CHL, the gene encoding the filament and stator was upregulated by CIP, and the whole assembly was upregulated by KAN (Fig. 2I and Fig. S33), suggesting improved mobility upon stress of these antibiotics. Three aspects of cell-environment communications were found to be differentially regulated (Fig. 2J and Fig. S34). Pilus (GO 0009289, pilus) formation was upregulated on CIP, TET, MMC, and KAN (Fig. 2H). Biofilm formation (GO 0042710, biofilm formation, and EcoCyc PWY-8243, colanic acid [Escherichia coli K-12] biosynthesis) was upregulated on CIP, MMC, and KAN (Fig. 2H and Fig. S33C). In contrast, quorum sensing (autoinducer 2 biosynthesis) was downregulated on CIP, TET, CAZ, IPM, MMC, and KAN.

### Transcriptomic comparison of physiological aspects impacted by antibiotics.

A transcriptomic comparison of physiological aspects impacted by antibiotic treatment is summarized in Fig. 2K. Similar to the analysis of the proportion of differentially regulated genes (Table 1), KAN impacted cell physiology the most, whereas PME had little impact on cell physiology. Different antibiotics led to shared and different impacts on E. coli’s transcriptomes. For carbon metabolism, stress response, amino acid biosynthesis, and quorum sensing, treatment with different antibiotics led to nearly the same downregulation. The responses in nucleotide biosynthesis, mobility, pilus, and biofilm were restricted to several antibiotics, although all these antibiotics led to the same upregulation. The antibiotics’ impact on energy conservation, protein biosynthesis, and cell wall biosynthesis were different: KAN regulated these cellular functions differently with the majority of antibiotics, and CHL also had a different regulatory pattern from those of ERY, CAZ, and IPM. KAN also led to a specific coupregulation of antibiotic resistance efflux pumps.

### Shared differentially regulated genes upon treatment with different antibiotics.

Genes that were coupregulated or codownregulated under treatment with all nine antibiotics were analyzed, leading to the finding of 8 coupregulated genes and 30 codownregulated genes (Table 2). Out of these 38 genes, 11 are stress response related and 18 are sulfur metabolism related. Therefore, these two aspects of physiology exhibited coregulation by treatment with antibiotics.

**TABLE 2:** Genes codifferentially regulated upon antibiotic treatment^*a*^

Gene	Gene ID	Fold change									Product name	Annotated function
		TET	MMC	IPM	CAZ	KAN	CIP	PME	ERY	CHL		
Upregulation												
b0721	*sdhC*	20.99	6.54	10.11	8.95	6.15	5.89	4.18	9.81	6.89	Succinate dehydrogenase	Respiration
b1530	*marR*	2.99	4.65	9.36	6.98	43.75	125.87	5.23	6.12	3.16	DNA-binding transcriptional repressor	Antibiotic resistance
b1558	*cspF*	26.55	10.75	6.23	9.30	5.45	13.73	5.09	8.45	5.03	Cold shock-like protein	Cold stress response
b1915	*yecF*	3.38	4.55	2.54	4.29	3.26	4.34	2.94	2.32	4.52	DUF2594 domain-containing protein	Unknown
b2533	*suhB*	2.48	9.52	6.48	7.37	4.78	8.04	2.45	3.16	1.92	Nus factor	rRNA maturation
b2667	*ygaV*	8.57	7.34	32.22	26.21	51.66	20.28	16.32	41.66	11.80	Putative DNA-binding transcriptional regulator	Transcription regulation
b2668	*ygaP*	8.89	9.77	36.46	31.32	25.78	13.63	12.52	12.49	7.68	Thiosulfate sulfurtransferase	Sulfur metabolism
b3704	*rnpA*	3.45	4.39	4.35	2.95	4.18	7.43	2.74	9.75	7.82	RNase P protein component	tRNA processing
Downregulation												
b0019	*nhaA*	4.05	1.87	2.06	1.78	2.45	3.23	2.36	4.66	5.32	Na^+^:H^+^ antiporter	Sodium ion homeostasis
b0207	*dkgB*	3.07	2.27	3.38	3.92	2.20	2.08	3.95	6.92	3.02	Methylglyoxal reductase	Detoxification
b0486	*ybaT*	175.04	15.06	4.98	15.49	49.96	21.34	2.77	2.49	4.34	Putative copper transporter	Copper resistance response
b0605	*ahpC*	8.14	4.61	2.67	4.24	15.62	5.01	3.69	7.07	5.19	Peroxidase component of alkyl hydroperoxide reductase	Oxidative stress response
b0606	*ahpF*	2.44	4.51	2.81	4.41	4.60	3.53	3.11	3.05	4.05	Peroxiredoxin reductase component of alkyl hydroperoxide reductase	Oxidative stress response
b0828	*iaaA*	3.39	6.49	4.05	4.13	4.11	4.29	2.89	3.26	2.33	Isoaspartyl dipeptidase proenzyme	Amino acid biosynthesis
b0953	*rmf*	35.74	5.48	3.41	4.10	35.20	44.40	5.92	17.44	6.48	Ribosome modulation factor	Ribosome protection
b1287	*yciW*	8.76	7.19	12.28	9.72	6.18	8.94	17.57	17.79	10.55	Putative peroxidase	Cysteine tolerance
b1729	*tcyP*	2.05	4.28	7.81	3.79	1.99	3.79	9.41	2.42	3.03	Cystine/sulfocysteine:cation symporter	Sulfur metabolism
b2013	*tsuA*	4.97	10.21	49.13	8.45	11.81	7.87	51.64	5.63	4.76	Thiosulfate transporter	Sulfur metabolism
b2135	*yohC*	9.91	5.04	4.95	5.19	37.95	15.41	2.01	4.93	8.82	Putative inner membrane protein	Unknown
b2241	*glpA*	10.13	10.14	28.37	22.93	4.99	4.83	35.01	4.24	23.38	Anaerobic glycerol-3-phosphate dehydrogenase subunit A	Respiration
b2414	*cysK*	2.72	8.25	12.56	6.50	20.56	10.22	9.73	6.19	4.77	Cysteine synthase A	Sulfur metabolism
b2421	*cysM*	2.57	4.57	4.37	4.20	3.91	4.26	5.51	3.81	3.82	Cysteine synthase B	Sulfur metabolism
b2422	*cysA*	13.77	27.91	34.32	15.51	16.44	39.86	67.64	23.07	7.29	Sulfate/thiosulfate ABC transporter ATP binding subunit	Sulfur metabolism
b2423	*cysW*	17.90	27.37	22.88	13.64	12.07	40.12	85.87	23.99	10.81	Sulfate/thiosulfate ABC transporter inner membrane subunit CysW	Sulfur metabolism
b2424	*cysU*	18.47	21.31	20.92	13.64	13.79	46.49	72.58	23.26	12.74	Sulfate/thiosulfate ABC transporter inner membrane subunit CysU	Sulfur metabolism
b2425	*cysP*	21.85	29.53	29.08	16.11	25.79	44.15	71.89	30.03	16.93	Thiosulfate/sulfate ABC transporter periplasmic binding protein CysP	Sulfur metabolism
b2750	*cysC*	6.62	12.11	20.88	11.62	5.31	14.38	13.37	18.23	6.20	Adenylyl-sulfate kinase	Sulfur metabolism
b2751	*cysN*	9.37	13.65	10.53	12.43	5.65	13.61	11.04	8.13	5.13	Sulfate adenylyltransferase subunit 1	Sulfur metabolism
b2752	*cysD*	11.02	20.49	39.05	17.08	10.65	21.38	41.75	24.31	16.82	Sulfate adenylyltransferase subunit 2	Sulfur metabolism
b2762	*cysH*	11.52	11.45	23.94	6.03	36.37	37.69	36.47	19.21	10.43	Phosphoadenosine phosphosulfate reductase	Sulfur metabolism
b2763	*cysI*	13.60	16.99	23.33	9.71	30.77	50.28	79.25	29.45	9.34	Sulfite reductase, hemoprotein subunit	Sulfur metabolism
b2764	*cysJ*	14.06	15.34	16.53	9.79	76.36	40.98	74.41	26.08	10.07	Sulfite reductase, flavoprotein subunit complex	Sulfur metabolism
b3491	*yhiM*	242.66	18.38	18.48	64.46	67.48	33.71	3.04	9.90	38.44	Inner membrane protein	Acid stress response
b3990	*thiH*	10.10	8.77	4.85	4.22	12.70	1.93	3.10	9.28	11.33	2-Iminoacetate synthase	Sulfur metabolism
b3991	*thiG*	10.16	8.62	4.87	4.37	8.92	1.92	2.99	6.17	8.21	1-Deoxy-d-xylulose 5-phosphate:thiol sulfurtransferase	Sulfur metabolism
b3992	*thiF*	9.47	6.95	5.12	4.32	8.25	1.63	3.07	11.80	11.96	Sulfur carrier protein, ThiS adenylyltransferase	Sulfur metabolism
b4452	*gadY*	5.50	3.22	4.14	6.97	7.14	2.14	3.55	4.25	14.11	Small regulatory RNA	Acid stress response
b4704	*arrS*	264.70	10.50	9.46	14.81	438.25	18.50	2.73	5.55	27.53	Small regulatory RNA	Acid stress response

a Upregulated fold change indicates the FPKMs of the antibiotic-treated group in relation to the control group. Downregulation fold change indicates the FPKMs of the control group in relation to the antibiotic-treated group. ID, identifier.

The *marR* gene, which is a global regulator of antibiotic resistance genes, was shown to be upregulated upon treatment with all antibiotics, in agreement with its important role in regulating antibiotic resistance. However, stress response genes for other stresses were differentially regulated. Other than the putatively cold stress response-related *cspF*, which was coupregulated, stress response genes for sodium ions, copper, oxidative stress, and acid stress were codownregulated. This suggests that regulation of these stress response pathways is stress specific, and this downregulation may help cells to better deal with antibiotics.

A significant finding with this analysis is the strong and shared codownregulation of all sulfur metabolism-related genes. This is in agreement with the finding that sulfur uptake and assimilation (Fig. 2C and Fig. S24A and S25) and biosynthesis of sulfur-containing amino acids (Fig. 2E and Fig. S29G and 29H) were downregulated after treatment with all antibiotics. This finding prompted us to wonder whether the response to antibiotics and sulfur metabolism are related in E. coli.

### Regulation of transcription factors.

**FIG 3:**
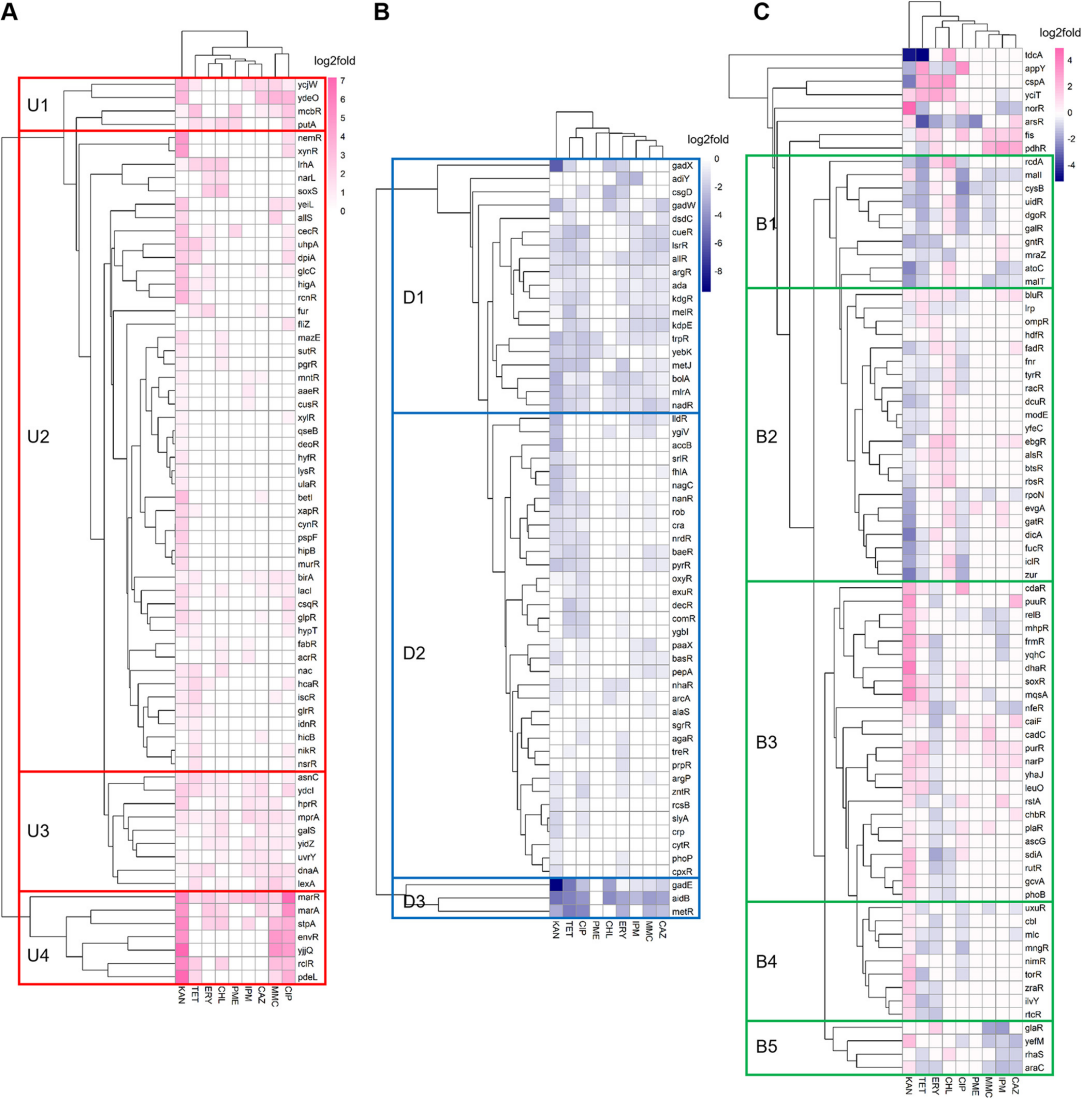
Regulation of transcription factors by antibiotics. (A) Upregulated transcription factors; (B) downregulated transcription factors; (C) bidirectionally regulated (both upregulated and downregulated) transcription factors.

A total of 210 transcription factors were found to be expressed in investigated transcriptomes (Fig. 3), out of which only 7 (*alpA*, *envY*, *feaR*, *rhaR*, *tdcR*, *gutM*, and *frlR*) were not responsive to antibiotics. Seventy-seven genes showed bidirectional regulation by different antibiotics, also known as upregulation on some antibiotics and downregulation on some others. KAN led to significant differential regulation of the majority of transcription factor-coding genes (172/210 [81.9%]), whereas PME led to significant differential regulation of very few transcription factor-coding genes (9/210 [4.3%]). This is in agreement with the fraction of all genes that were significantly regulated by these two antibiotics (Table 1).

With hierarchal clustering analysis, the transcription factors could be clustered in coregulated groups suggesting shared regulatory patterns. For genes that were upregulated by antibiotics (Fig. 3A), four clusters could be observed: cluster U1 and U3 genes showed general upregulation on most antibiotics except for PME, cluster U2 genes were upregulated by KAN, and cluster U4 genes were strongly upregulated by KAN, MMC, and CIP. For genes that were downregulated by antibiotics (Fig. 3B), three clusters could be observed: cluster D1 genes were downregulated on most antibiotics, cluster D2 genes were commonly downregulated by KAN, TET, and CIP, and cluster D3 genes were strongly downregulated on most antibiotics except for PME. For genes that showed bidirectional regulation (Fig. 3C), five clusters could be observed: cluster B1 was characterized by upregulation on CHL and downregulation on KAN, TET, CIP, and MMC; cluster B2 was characterized by upregulation on ERY and CHL and downregulation on KAN, TET, and CIP; cluster B3 was characterized by upregulation on KAN, TET, and CIP and downregulation on ERY; cluster B4 was characterized by upregulation on KAN and downregulation on TET, ERY, and CIP; and cluster B5 was characterized by downregulation on MMC, IPM, and CAZ. This clustering pattern implies the presence of mechanisms for coordinated regulation of transcription factors which, in turn, govern global transcription regulation.

## DISCUSSION

Transcriptomics is a commonly used powerful tool to elucidate genome-wide changes of cellular functions in response to external or internal signals, substrates, and other disturbances. Although downstream protein translation efficiencies, posttranslational modifications, regulation of protein activities, and other cellular processes can also impact or even determine cellular functions, regulation at the transcriptional level nevertheless is still a major driver for regulation of cell physiology. Therefore, determining transcriptomic changes is a key step in understanding how cell function is modulated.

This work provides a comprehensive comparative analysis on how different antibiotics impact bacterial transcriptomes using E. coli K-12 MG1655 as a model organism. Understanding how antibiotics impact bacterial physiology helps us in understanding how bacteria adapt to stresses from our most powerful weapons against bacterial infections, what bacteria can potentially do to resist antibiotics, and what may be a good target for further developing antibiotics. It also helps us better understand fundamental microbial processes. With this understanding, bacterial weaknesses against different classes of antibiotics may be exposed, which may provide hints on how the synergistic use of antibiotics could target different weaknesses and show higher efficacy.

Treatment with antibiotics led to enormous changes in the transcriptomes of E. coli. The high proportions of differentially expressed genes, enriched pathways, and GO terms all indicate that antibiotics have in general substantial impacts on E. coli’s physiology. This is also supported by further detailed analysis showing that nearly all major aspects of cell physiology are influenced by antibiotics. On the other hand, the level of impacts differs from antibiotic to antibiotic. Judging from the proportion of the genome that is significantly up- or downregulated, KAN appears to have the strongest impact, whereas the impact of PME appears minor. This observation suggests that antibiotics do not elicit the same responses in E. coli.

With in-depth comparison of transcriptomic changes following treatment of each antibiotic, we were able to summarize the major aspects of physiology impacted by antibiotics, and compare changes between antibiotics (Fig. 2K). The global downregulation of carbon metabolism, stress response (except for antibiotic resistance and SOS response), amino acid biosynthesis, and quorum sensing suggest common strategies against these antibiotics: cells reduce food hunting, shut down unnecessary response mechanisms, stop energy-consuming processes, and cease communication and intercellular collaborations. These responses suggest acute passive reactions of E. coli to step back from routine business and save for harsh times. Meanwhile, in response to certain antibiotics, including CHL, CIP, ERY, TET, MMC, and KAN (antibiotics that are not cell wall inhibitors like CAZ, IPM, and PME), E. coli upregulates proactive processes to save itself: it upregulates mobility and pilus to evade detrimental chemicals and upregulates pilus and biofilm formation to go into a more protective form. The SOS response, which is a key stress response pathway, is activated upon treatment of antibiotics that interfere with DNA replication, which agrees with the mechanism of the SOS response: it responds to accumulation of single-stranded DNA and initiates DNA repair (31). With other antibiotics, SOS response pathways are not always activated, with upregulation observed only on CIP, TET, CAZ, and KAN. For energy conservation, protein biosynthesis, and cell wall biosynthesis, E. coli adopts different strategies for KAN and for other antibiotics. For KAN, E. coli shuts down energy conservation, protein biosynthesis, and cell wall biosynthesis, whereas for other antibiotics, E. coli upregulates or does not change its energy conservation, upregulates protein biosynthesis and nucleotide biosynthesis (possibly for the synthesis of proteins needed to respond to antibiotics), and reinforces the cell wall (mostly for cell wall inhibitors, not for CHL). This fundamental difference may suggest that E. coli considers KAN a major threat and shuts down everything that is not needed, whereas for the other antibiotics, E. coli is still getting ready for a battle. In agreement with this, other than antibiotic-specific β-lactam and polymyxin resistance pathways, E. coli fully upregulates its arsenal of efflux pumps in response to KAN (Fig. S25), to proactively extrude this antibiotic from its cytoplasm.

**FIG 4:**
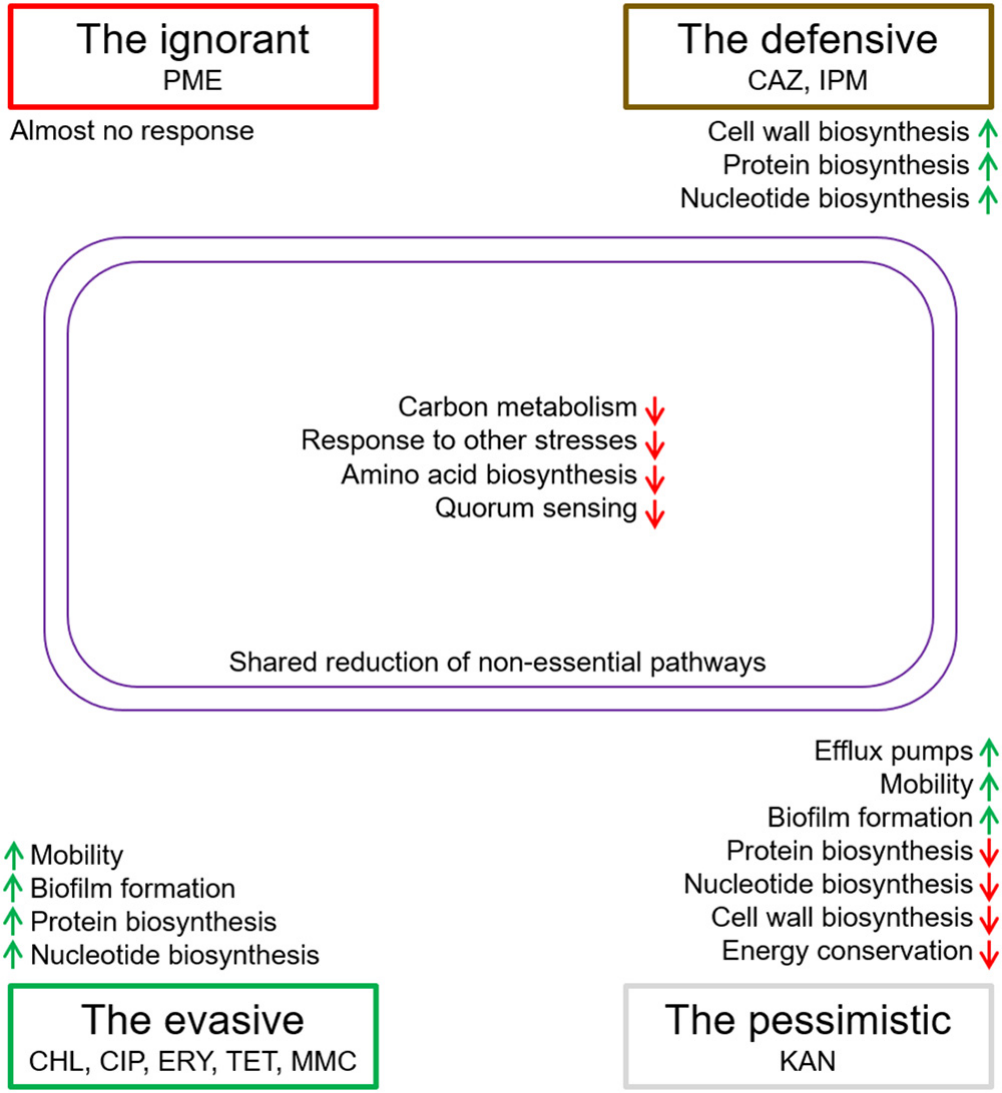
Shared and unique strategies E. coli uses against antibiotic stress.

These transcriptomic analyses paint us a picture of how E. coli uses different strategies against different antibiotics. E. coli considers KAN a highly dangerous antibiotic and therefore shuts down everything possible, gives up active adaptation, proactively turns on efflux pumps, initiates movements, and gets ready to form more protective biofilms. In this case, E. coli plays a pessimistic game, to hibernate, run, and defend. For the other antibiotics, E. coli believes it can still put up a fight and therefore upregulates protein and nucleotide biosynthesis and gets ready to synthesize whatever protein may be needed to proactively adapt to threat. Meanwhile, when dealing with cell wall inhibitors, E. coli is more defensive, as it upregulates cell wall biosynthesis in an attempt to overcome the weakening of the cell wall; when dealing with non-cell wall inhibitors, E. coli is more evasive, as E. coli upregulates mobility and biofilm formation; and when dealing with PME, E. coli is ignorant, as it almost does not change its transcriptome. Although there are still other antibiotic-specific responses, we believe that we can classify the strategies E. coli uses against antibiotics into four categories: the pessimistic (KAN), the defensive (CAZ and IPM), the evasive (CHL, CIP, ERY, TET, and MMC), and the ignorant (PME). A summary of this theory is shown in Fig. 4. With Uniform Manifold Approximation and Projection (UMAP) analysis, it can be further observed that the transcriptomes of each category appear to cluster together, with the exception of MMC, supporting the general suggestion that E. coli applies different strategies for dealing with antibiotic stress.

Although E. coli apparently uses different strategies for different antibiotics in accordance to the level of threat it probes, the levels of threat from all nine antibiotics are actually the same (50% inhibited growth). We believe that the stronger the response is, the most likely cells can successfully survive under the same level of threat. Therefore, out of all nine antibiotics, KAN is possibly the least dangerous, although E. coli is more pessimistic. Meanwhile, the most dangerous chemical out of all antibiotics, we believe, is PME, as it leads to little transcriptomic response, although it has the same level of threat as the other antibiotics. This may have to do with the particularly good efficacy of PME against all Gram-negative bacteria and the fact that it evades most antibiotic resistance mechanisms.

In conclusion, the transcriptomic responses of E. coli to nine classes of antibiotics were studied in detail. Large changes in transcriptomes were found, suggesting that the impact of antibiotics on E. coli’s physiology is significant. E. coli uses both shared and unique strategies against the stress of antibiotics, and several interesting findings, including the global downregulation of sulfur metabolism, were obtained.

## MATERIALS AND METHODS

### Bacterial strain and antibiotics.

E. coli K-12 strain MG1655 was used as a model system to analyze the dynamic transcriptional response to subinhibitory concentrations of antibiotics.

Nine different classes of antibiotics were used in this work: tetracycline (TET; tetracyclines), mitomycin C (MMC; mitomycins), imipenem (IPM; carbapenems), ceftazidime (CAZ; β-lactams), kanamycin (KAN; aminoglycosides), ciprofloxacin (CIP; quinolones), polymyxin E (PME; colistins), erythromycin (ERY; macrolides), and chloramphenicol (CHL; amphenicols). The concentrations of antibiotics used in this study are listed in Table S1 in the supplemental material. Erythromycin and chloramphenicol were dissolved in 100% ethanol. All seven other antibiotics were dissolved in sterile double-distilled water.

### Determination of bacterial growth curves.

E. coli K-12 strain MG1655 was grown to mid-logarithmic phase in LB broth at 37°C, diluted 100-fold, and inoculated to 96-well microplates containing 300 μL of Mueller-Hinton broth (MHB) medium with different concentrations of antibiotics in each well. Each antibiotic concentration was repeated in three wells. The microplates were incubated at 37°C with shaking at 200 rpm for 24 h, and the value of optical density at 600 nm (OD_600_) was measured every 1 h using the Automated Microbiology Growth Analysis System FP-1100-C (Bioscreen, Finland). The growth curves were modeled by fitting the dose-response model shown in equation 1:

(1) $$y=A_{1}+\frac{A_{2}-A_{1}}{1+10^{\log(x_{0}-x)\times p}}$$

In equation 1, *y* represents value of OD_600_, *x* represents the growth time, and *A*_1_, *A*_2_, *p*, and *x*_0_ are constants.

### Determination of IC_50_.

Based on growth curves, the concentration-inhibition rate curves were modeled by fitting the logistic model shown in equation 2:

(2) $$y=A_{2}+\frac{A_{1}-A_{2}}{1+{\left(\frac{x}{x_{0}}\right)}^{p}}$$

In equation 2, *y* represents percent growth inhibition, *x* represents concentration of antibiotics, and *A*_1_, *A*_2_, *p*, and *x*_0_ are constants (32). When *y* equals 50%, the *x* value calculated with equation 2 is the IC_50_ value indicating the level of antibiotic at 50% inhibition (33).

### Bacterial growth and RNA extraction.

E. coli K-12 strain MG1655 was cultivated in LB broth at 37°C and grown to mid-logarithmic phase. Then the culture was inoculated in MHB medium (1% inoculant) containing IC_50_ values of antibiotics and grown to mid-logarithmic phase with shaking (Table S1). Three replicates were performed for each antibiotic. Cultures with addition of 100% ethanol in place of antibiotics were used as controls for erythromycin and chloramphenicol. For the other seven antibiotics, cultures with no antibiotic or ethanol addition were used as controls. RNA was extracted using the bacterial RNA kit (Omega Bio-Tek, USA) according to the manufacturer’s instructions.

### Transcriptome sequencing and bioinformatics.

A total amount of 3 μg of RNA per sample was used for transcriptomic analysis. Sequencing libraries were generated using NEBNext Ultra directional RNA library prep kit. Sequencing was done with Illumina HiSeq 2500. Read mapping was performed using Bowtie2 (34). HTSeq was used for quantification of expression levels (35). DESeq on the R platform was used for calculation of differentially expressed genes (36). GO, KEGG, and EcoCyc enrichment analysis was done with Fisher’s exact test. All *P* values were adjusted with the Benjamini-Hochberg procedure to generate false-discovery rates (FDRs; adjusted *P* values). GO enrichment analysis was performed using the clusterProfiler package on the R platform (37). The E. coli database used for mapping was org.EcK12.eg.db. GO clustering was done with the emapplot function of the clusterProfiler package. EcoCyc enrichment analysis was done with the enrichment tools in the SmartTable functions on the EcoCyc website (38). KEGG pathway mapping and figure generation were performed with the PathView website (39). For hierarchal clustering analysis of gene expression, the gene expression levels were normalized to z-scores of log_10_(FPKM + 1), where FPKM is fragments per kilobase per million. For hierarchal clustering analysis of the regulation of transcription factors, the UMPGA method was used as the clustering method. Transcription of genes that were not significantly regulated was considered unchanged. The transcription factor list was obtained from RegulonDB (40). Hierarchical clustering analysis and heat map calculation were performed with the pheatmap package on R platform. For UMAP analysis of FPKM vectors of each transcriptome, the umap package on R platform was used. The ggplot2 package was used for figure drawing.

### Data availability.

Transcriptomic data from this work can be found at Gene Expression Omnibus under accession number GSE220559.
