# Supplementary material for: Comparative Analysis of Transcriptomic Response of Escherichia coli K-12 MG1655 to Nine Representative Classes of Antibiotics
Source: Microbiol Spectr. 2023 Feb 28;11(2):e00317-23. doi: 10.1128/spectrum.00317-23 (PMC10100721; doi:10.1128/spectrum.00317-23)
Supplement: Supplemental file 1 — Supplemental material. Download spectrum.00317-23-s0001.pdf, PDF file, 6.3 MB [file spectrum.00317-23-s0001.pdf]

Supplementary materials

Table S1. Concentrations of antibiotics used in this study.

Antibiotic	Concentration (μg/mL)
Tetracycline	1.8
Mitomycin C	1.4
Imipenem	0.06
Ceftazidime	0.12
Kanamycin	1.6
Ciprofloxacin	0.012
Polymyxin E	0.4
Chloramphenicol	1.1
Erythromycin	7.0

Table S2. Read counts, FPKMs, annotations, and differential gene expression of the transcriptomes.

Table S3. Enriched KEGG pathways after antibiotic treatment. *p*-values indicate adjusted *p*-values with the Benjamini-Hochberg procedure.

**Tetracycline**			
**Upregulated KEGG pathways**		**Downregulated KEGG pathways**	
KEGG pathways	*p*-value	KEGG pathways	*p*-value
Ribosome	1.60×10^{-12}	Glycolysis / Gluconeogenesis	3.15×10^{-4}
Valine, leucine and isoleucine biosynthesis	1.08×10^{-3}	Histidine metabolism	2.60×10^{-3}
C5-Branched dibasic acid metabolism	0.023	Selenocompound metabolism	2.60×10^{-3}
Oxidative phosphorylation	0.039	Microbial metabolism in diverse environments	4.63×10^{-3}
		Quorum sensing	6.60×10^{-3}
		Sulfur metabolism	0.013
		Biosynthesis of amino acids	0.014
		ABC transporters	0.015

**Mitomycin C**			
**Upregulated KEGG pathways**		**Downregulated KEGG pathways**	
KEGG pathways	*p*-value	KEGG pathways	*p*-value
Biosynthesis of cofactors	0.015	Biosynthesis of secondary metabolites	3.95×10^{-6}
		ABC transporters	2.59×10^{-5}
		Histidine metabolism	3.08×10^{-4}
		Valine, leucine and isoleucine biosynthesis	4.24×10^{-3}
		Monobactam biosynthesis	5.47×10^{-3}
		Cysteine and methionine metabolism	9.48×10^{-3}
		Sulfur metabolism	0.037
		2-Oxocarboxylic acid metabolism	0.044

**Imipenem**			
**Upregulated KEGG pathways**		**Downregulated KEGG pathways**	
KEGG pathways	*p*-value	KEGG pathways	*p*-value
Ribosome	1.01×10^{-9}	Biosynthesis of amino acids	3.46×10^{-5}
Purine metabolism	1.06×10^{-4}	Histidine metabolism	3.46×10^{-5}
Biosynthesis of cofactors	2.80×10^{-4}	ABC transporters	7.89×10^{-4}
Peptidoglycan biosynthesis	0.011	Sulfur metabolism	1.30×10^{-3}
Sulfur relay system	0.029	Fructose and mannose metabolism	2.95×10^{-3}
Nucleotide metabolism	0.032	Phosphotransferase system	6.13×10^{-3}

		(PTS)	
RNA degradation	0.032	Glycolysis / Gluconeogenesis	0.017
		Pyruvate metabolism	0.039
		Amino sugar and nucleotide sugar metabolism	0.039
**Ceftazidime**			
**Upregulated KEGG pathways**		**Downregulated KEGG pathways**	
KEGG pathways	*p*-value	KEGG pathways	*p*-value
beta-Lactam resistance	1.07×10^{-7}	Biosynthesis of amino acids	3.80×10^{-8}
Purine metabolism	7.90×10^{-4}	Biosynthesis of secondary metabolites	3.49×10^{-5}
Biosynthesis of cofactors	3.61×10^{-3}	Histidine metabolism	3.49×10^{-5}
Nucleotide metabolism	0.017	Sulfur metabolism	0.010
		Valine, leucine and isoleucine biosynthesis	0.012
		Starch and sucrose metabolism	0.012
		2-Oxocarboxylic acid metabolism	0.047
**Kanamycin**			
**Upregulated KEGG pathways**		**Downregulated KEGG pathways**	
KEGG pathways	*p*-value	KEGG pathways	*p*-value
Flagellar assembly	1.94×10^{-8}	Biosynthesis of secondary metabolites	1.19×10^{-9}
Two-component system	5.40×10^{-8}	Oxidative phosphorylation	1.31×10^{-8}
Bacterial chemotaxis	8.48×10^{-4}	Carbon metabolism	4.62×10^{-7}
Pentose and glucuronate interconversions	0.022	Biosynthesis of amino acids	1.53×10^{-4}
		Glycolysis / Gluconeogenesis	2.30×10^{-3}
		Glutathione metabolism	2.30×10^{-3}
		Pyruvate metabolism	4.50×10^{-3}
		Biosynthesis of cofactors	7.53×10^{-3}
		Microbial metabolism in diverse environments	8.86×10^{-3}
		Citrate cycle (TCA cycle)	0.025
		Cysteine and methionine metabolism	0.025
		Alanine, aspartate and glutamate metabolism	0.032
**Ciprofloxacin**			
**Upregulated KEGG pathways**		**Downregulated KEGG pathways**	
KEGG pathways	*p*-value	KEGG pathways	*p*-value
Bacterial chemotaxis	2.27×10^{-3}	Biosynthesis of secondary metabolites	3.28×10^{-9}

Ribosome	0.028	Biosynthesis of amino acids	6.56×10^{-9}
O-Antigen nucleotide sugar biosynthesis	0.045	Glutathione metabolism	5.97×10^{-4}
Bacterial secretion system	0.049	Carbon metabolism	6.21×10^{-4}
		Histidine metabolism	8.71×10^{-4}
		Microbial metabolism in diverse environments	3.14×10^{-3}
		Glycolysis / Gluconeogenesis	3.64×10^{-3}
		Selenocompound metabolism	4.56×10^{-3}
		Oxidative phosphorylation	4.56×10^{-3}
		Sulfur metabolism	4.85×10^{-3}
		Pyruvate metabolism	0.011
		Phenylalanine, tyrosine and tryptophan biosynthesis	0.012
		Cysteine and methionine metabolism	0.013
		Quorum sensing	0.018
		Glycine, serine and threonine metabolism	0.025
**Polymyxin E**			
**Upregulated KEGG pathways**		**Downregulated KEGG pathways**	
KEGG pathways	*p*-value	KEGG pathways	*p*-value
Cationic antimicrobial peptide (CAMP) resistance	0.017	Biosynthesis of siderophore group nonribosomal peptides	1.13×10^{-4}
Amino sugar and nucleotide sugar metabolism	0.023	ABC transporters	2.41×10^{-3}
		Thiamine metabolism	2.41×10^{-3}
		Monobactam biosynthesis	0.028
**Chloramphenicol**			
**Upregulated KEGG pathways**		**Downregulated KEGG pathways**	
KEGG pathways	*p*-value	KEGG pathways	*p*-value
Oxidative phosphorylation	6.11×10^{-6}	Sulfur metabolism	0.011
Nitrogen metabolism	4.52×10^{-5}	Biosynthesis of siderophore group nonribosomal peptides	0.013
Sulfur relay system	3.37×10^{-4}	Biosynthesis of secondary metabolites	0.013
		Glycolysis / Gluconeogenesis	0.036
		Biosynthesis of amino acids	0.036
**Erythromycin**			
**Upregulated KEGG pathways**		**Downregulated KEGG pathways**	
KEGG pathways	*p*-value	KEGG pathways	*p*-value
Oxidative phosphorylation	4.02×10^{-7}	Glycolysis / Gluconeogenesis	3.02×10^{-4}
Nitrogen metabolism	5.88×10^{-5}	Sulfur metabolism	3.02×10^{-4}

Histidine metabolism	9.65×10^{-4}	Biosynthesis of secondary metabolites	1.60×10^{-3}
Ribosome	4.20×10^{-3}	Microbial metabolism in diverse environments	5.25×10^{-3}
Sulfur relay system	0.021	Pyruvate metabolism	0.021
Mismatch repair	0.026	Arginine biosynthesis	0.022
Valine, leucine and isoleucine biosynthesis	0.045	Cysteine and methionine metabolism	0.047
		Starch and sucrose metabolism	0.047
		Alanine, aspartate and glutamate metabolism	0.047

Table S4. Enriched Ecocyc pathways after antibiotic treatment. *p*-values indicate adjusted *p*-values with the Benjamini-Hochberg procedure.

<table>
<tr><th colspan="4">Tetracycline</th></tr>
<tr><th colspan="2">Upregulated Ecocyc pathways</th><th colspan="2">Downregulated Ecocyc pathways</th></tr>
<tr><td>Ecocyc pathways</td><td>p-value</td><td>Ecocyc pathways</td><td>p-value</td></tr>
<tr><td>ATP biosynthesis</td><td>$6.80×10^{-4}$</td><td>Degradation/Utilization/Assimilation</td><td>0.021</td></tr>
<tr><td>Nucleoside and nucleotide biosynthesis</td><td>$6.50×10^{-3}$</td><td>Generation of precursormetabolites and energy</td><td>0.021</td></tr>
<tr><td>Succinate to cytochrome bo oxidase electron transfer</td><td>$6.50×10^{-3}$</td><td>L-histidine biosynthesis</td><td>0.021</td></tr>
<tr><td>Purine nucleotide de novo biosynthesis</td><td>$6.50×10^{-3}$</td><td>L-histidine biosynthesis</td><td>0.021</td></tr>
<tr><td>Superpathway of purine nucleotides de novo biosynthesis II</td><td>$6.50×10^{-3}$</td><td>Nitrate reduction VIII (dissimilatory)</td><td>0.028</td></tr>
<tr><td>Purine nucleotide biosynthesis</td><td>$9.80×10^{-3}$</td><td>Hydrogen to dimethyl sulfoxide electron transfer</td><td>0.031</td></tr>
<tr><td>N-acetylneuraminate and N-acetylmannosamine degradation I</td><td>0.015</td><td>Electron transfer chains</td><td>0.04</td></tr>
<tr><td>N-Acetylneuraminate and N-acetylmannosamine degradation</td><td>0.015</td><td></td><td></td></tr>
<tr><td>Inosine-5'-phosphate biosynthesis</td><td>0.015</td><td></td><td></td></tr>
<tr><td>Inosine-5'-phosphate biosynthesis I</td><td>0.015</td><td></td><td></td></tr>
<tr><td>Superpathway of enterobacterial common antigen biosynthesis</td><td>0.016</td><td></td><td></td></tr>
<tr><th colspan="4">Mitomycin C</th></tr>
<tr><th colspan="2">Upregulated Ecocyc pathways</th><th colspan="2">Downregulated Ecocyc pathways</th></tr>
<tr><td>Ecocyc pathway</td><td>p-value</td><td>Ecocyc pathway</td><td>p-value</td></tr>
<tr><td>Nucleic acid processing</td><td>$3.62×10^{-3}$</td><td>Proteinogenic amino acid biosynthesis</td><td>$2.64×10^{-10}$</td></tr>
<tr><td>Nucleoside and nucleotide biosynthesis</td><td>$7.74×10^{-3}$</td><td>Amino acid biosynthesis</td><td>$7.04×10^{-10}$</td></tr>
<tr><td>Superpathway of pyrimidine ribonucleotides de novo biosynthesis</td><td>$7.82×10^{-3}$</td><td>L-histidine biosynthesis</td><td>$2.05×10^{-3}$</td></tr>
</table>

Pyrimidine ribonucleotide *de novo* biosynthesis	7.82×10^{-3}	Proteinogenic amino acid degradation	3.01×10^{-3}
UMP biosynthesis	0.023	Amino acid degradation	3.45×10^{-3}
UMP biosynthesis I	0.023	Alcohol degradation	5.06×10^{-3}
Purine riboucleotide *de novo* biosynthesis	0.023	NADH to cytochrome *bd* oxidase electron transfer I	5.06×10^{-3}
Superpathway of histidine, purine, and pyrimidine biosynthesis	0.031	Degradation/Utilization/Assimi lation	8.68×10^{-3}
		NADH to hydrogen peroxide electron transfer	0.017
		NADH to trimethylamine *N*-oxide electron transfer	0.036
		S-adenosyl-L-methionine biosynthesis	0.046
		L-tryptophan biosynthesis	0.046
		L-tryptophan biosynthesis	0.046
		Superpathway of *S*-adenosyl-L-methionine biosynthesis	0.046
		NADH to dimethyl sulfoxide electron transfer	0.046
		L-leucine biosynthesis	0.046
		L-threonine biosynthesis	0.046
		L-leucine biosynthesis	0.046
		Superpathway of L-threonine biosynthesis	0.046
**Imipenem**			
**Upregulated Ecocyc pathways**		**Downregulated Ecocyc pathways**	
Ecocyc pathway	*p*-value	Ecocyc pathway	*p*-value
Nucleoside and nucleotide biosynthesis	6.31×10^{-5}	Detoxification	5.71×10^{-5}
Nucleic acid processing	6.31×10^{-5}	Proteinogenic amino acid biosynthesis	5.71×10^{-5}
Purine nucleotide biosynthesis	2.30×10^{-4}	Degradation/Utilization/Assimi lation	5.71×10^{-5}
Purine riboucleotide *de novo* biosynthesis	3.54×10^{-3}	Alcohol degradation	8.96×10^{-5}
Superpathway of histidine, purine, and pyrimidine biosynthesis	3.54×10^{-3}	Amino acid biosynthesis	8.96×10^{-5}
Purine nucleotide *de novo* biosynthesis	3.54×10^{-3}	L-histidine biosynthesis	8.96×10^{-5}

Superpathway of purine nucleotides *de novo* biosynthesis II	3.54×10^{-3}	Glycerol degradation	5.10×10^{-4}
tRNA-uridine 2-thiolation and selenation (bacteria)	5.21×10^{-3}	Glycerol and glycerophosphodiester degradation	0.016
Inosine-5'-phosphate biosynthesis	5.68×10^{-3}	Glycolysis I (from glucose 6-phosphate)	0.024
Inosine-5'-phosphate biosynthesis I	5.68×10^{-3}	L-lysine degradation	0.024
Macromolecule modification	0.039	L-lysine degradation I	0.024
		Superpathway of glycolysis and the Entner-Doudoroff pathway	0.027
		Glycolysis	0.031
		Assimilatory sulfate reduction	0.031
		Assimilatory sulfate reduction I	0.031
		Superpathway of sulfate assimilation and cysteine biosynthesis	0.037
		Superoxide radicals degradation	0.037
		Reactive oxygen species degradation	0.037
		Glycerophosphodiester degradation	0.037
		Proteinogenic amino acid Degradation	0.037
**Ceftazidime**			
**Upregulated Ecocyc pathways**		**Downregulated Ecocyc pathways**	
Ecocyc pathway	*p*-value	Ecocyc pathway	*p*-value
Nucleoside and nucleotide biosynthesis	8.74×10^{-6}	Proteinogenic amino acid biosynthesis	6.08×10^{-7}
Superpathway of histidine, purine, and pyrimidine biosynthesis	3.28×10^{-4}	Amino acid biosynthesis	8.74×10^{-7}
Purine nucleotide biosynthesis	3.28×10^{-4}	L-histidine biosynthesis	3.21×10^{-4}
Nucleic acid processing	3.75×10^{-4}	Superpathway of *N*-acetylglucosamine, *N*-acetylmannosamine and *N*-acetylneuraminate degradation	0.048
Purine nucleotide *de novo*	1.35×10^{-3}		

biosynthesis			
Superpathway of purine nucleotides *de novo* biosynthesis II	1.35×10^{-3}		
Purine riboucleotide *de novo* biosynthesis	0.015		
Superpathway of pyrimidine deoxyribonucleotides *de novo* biosynthesis	0.023		
Superpathway of pyrimidine ribonucleotides *de novo* biosynthesis	0.023		
Pyrimidine ribonucleotide *de novo* biosynthesis	0.023		
Pyrimidine nucleotide biosynthesis	0.039		
**Kanamycin**			
**Upregulated Ecocyc pathways**		**Downregulated Ecocyc pathways**	
Ecocyc pathway	*p*-value	Ecocyc pathway	*p*-value
Colanic acid (*Escherichia coli* K12) biosynthesis	0.010	Nitrate reduction VIII (dissimilatory)	2.85×10^{-4}
		NADH to cytochrome *bo* oxidase electron transfer I	6.68×10^{-4}
		NADH to fumarate electron transfer	6.68×10^{-4}
		NADH to dimethyl sulfoxide electron transfer	1.15×10^{-3}
		Superpathway of glycolysis, pyruvate dehydrogenase, TCA, and glyoxylate bypass	0.013
		Aerobic respiration	0.031
		NADH to hydrogen peroxide electron transfer	0.048
		NADH to cytochrome *bd* oxidase electron transfer I	0.048
		Lipid A-core biosynthesis (*E. coli* K-12)	0.048
		Lipid A-core biosynthesis	0.048
		Nitrogen compound metabolism	0.048
**Ciprofloxacin**			
**Upregulated Ecocyc pathways**		**Downregulated Ecocyc pathways**	
Ecocyc pathway	*p*-value	Ecocyc pathway	*p*-value

Superpathway of enterobacterial common antigen biosynthesis	2.45×10^{-3}	NADH to fumarate electron transfer	9.62×10^{-7}
Superpathway of D-glucarate and D-galactarate degradation	0.020	NADH to dimethyl sulfoxide electron transfer	1.67×10^{-6}
Glycolate degradation	0.020	NADH to cytochrome *bd* oxidase electron transfer I	1.38×10^{-5}
Polysaccharide biosynthesis	0.026	NADH to hydrogen peroxide electron transfer	3.55×10^{-4}
D-glucarate degradation I	0.031	NADH to trimethylamine *N*-oxide electron transfer	1.53×10^{-3}
D-glucarate degradation	0.031	Amino acid Biosynthesis	1.53×10^{-3}
D-galactarate degradation	0.031	Proteinogenic amino acid biosynthesis	5.64×10^{-3}
D-galactarate degradation I	0.031	*S*-adenosyl-L-methionine biosynthesis	5.70×10^{-3}
Glycolate and glyoxylate degradation I	0.031	L-histidine biosynthesis	6.60×10^{-3}
Superpathway of pyrimidine ribonucleotides *de novo* biosynthesis	0.036	NADH to cytochrome *bo* oxidase electron transfer I	8.60×10^{-3}
Pyrimidine ribonucleotide *de novo* Biosynthesis	0.036	Generation of precursor metabolites and energy	0.011
		Cardiolipin biosynthesis	0.016
		Superpathway of sulfate assimilation and cysteine biosynthesis	0.016
		Superpathway of glycolysis, pyruvate dehydrogenase, TCA, and glyoxylate bypass	0.016
		Superpathway of glycolysis and the Entner-Doudoroff pathway	0.018
		Detoxification	0.018
		Glycolysis I (from glucose 6-phosphate)	0.022
		Superpathway of *S*-adenosyl-L-methionine biosynthesis	0.023
		Aerobic respiration	0.027
		Nitrate reduction VIII (dissimilatory)	0.028
		Methylglyoxal detoxification	0.028
		Superpathway of	0.028

		methylglyoxal degradation	
		Glycolysis	0.028
		Cardiolipin biosynthesis I	0.028
		Methylglyoxal degradation III	0.028
		L-methionine biosynthesis I	0.028
		Proteinogenic amino acid degradation	0.041
		L-methionine *de novo* biosynthesis	0.041
		Assimilatory sulfate reduction	0.041
		L-methionine biosynthesis	0.041
		Assimilatory sulfate reduction I	0.041
		Superpathway of L-homoserine and L-methionine biosynthesis	0.041
		Aspartate superpathway	0.047
		Aldehyde degradation	0.047
		Amino acid degradation	0.047
**Polymyxin E**			
**Upregulated Ecocyc pathways**		**Downregulated Ecocyc pathways**	
Ecocyc pathway	*p*-value	Ecocyc pathway	*p*-value
Antibiotic Resistance	7.69×10^{-3}	Superpathway of sulfate assimilation and cysteine biosynthesis	1.78×10^{-5}
Polymyxin resistance	7.69×10^{-3}	Assimilatory sulfate reduction	3.39×10^{-5}
		Assimilatory sulfate reduction I	3.39×10^{-5}
		Inorganic nutrient metabolism	3.39×10^{-5}
		Sulfur compound metabolism	2.91×10^{-4}
		Metabolite Activation/ Inactivation/Interconversion	9.34×10^{-3}
		Sulfate activation for sulfonation	9.34×10^{-3}
		Metabolite activation	9.34×10^{-3}
		Superpathway of thiamine diphosphate biosynthesis I	9.34×10^{-3}
		Thiamine biosynthesis	0.015
		Siderophore and metallophore biosynthesis	0.015
		UMP Biosynthesis	0.015
		Enterobactin biosynthesis	0.015
		UMP biosynthesis I	0.015
		Molybdenum cofactor	0.039

		biosynthesis	
**Chloramphenicol**			
**Upregulated Ecocyc pathways**		**Downregulated Ecocyc pathways**	
Ecocyc pathway	*p*-value	Ecocyc pathway	*p*-value
Electron transfer chains	2.15×10^{-10}	Aminoacyl-tRNA charging	3.49×10^{-6}
Respiration	1.16×10^{-9}	tRNA charging	3.49×10^{-6}
Nitrate reduction	2.01×10^{-7}	Metabolic clusters	2.53×10^{-4}
Generation of precursor metabolites and energy	1.13×10^{-6}	Lipid A-core biosynthesis (*E. coli* K-12)	2.44×10^{-3}
Anaerobic respiration	5.97×10^{-6}	Lipid A-core biosynthesis	2.44×10^{-3}
Nitrogen compound metabolism	9.50×10^{-6}	Assimilatory sulfate reduction	2.44×10^{-3}
Aerobic respiration	2.07×10^{-5}	Assimilatory sulfate reduction I	2.44×10^{-3}
Succinate to cytochrome *bo* oxidase electron transfer	1.04×10^{-4}	Superpathway of sulfate assimilation and cysteine biosynthesis	6.86×10^{-3}
Nitrate reduction VIII (dissimilatory)	1.11×10^{-4}	Glycerol and glycerophosphodiester degradation	0.010
Nitrate reduction III (dissimilatory)	1.11×10^{-4}	Glycerol degradation I	0.010
NADH to cytochrome *bo* oxidase electron transfer I	1.59×10^{-4}	Cell structure biosynthesis	0.020
Adenosine nucleotide degradation	2.58×10^{-3}	Glycerol degradation	0.038
Formate to nitrite electron transfer	9.97×10^{-3}	Glycerophosphodiester degradation	0.042
D-lactate to cytochrome *bo* oxidase electron transfer	0.011		
Proline to cytochrome *bo* oxidase electron transfer	0.011		
Molybdopterin biosynthesis	0.011		
Inorganic nutrient metabolism	0.018		
Adenosine nucleotides degradation II	0.027		
NADH to dimethyl sulfoxide electron transfer	0.027		
ATP biosynthesis	0.030		
Purine nucleotide Degradation	0.040		
NADH to hydrogen peroxide electron transfer	0.043		
**Erythromycin**			

**Upregulated Ecocyc pathways**		**Downregulated Ecocyc pathways**	
Ecocyc pathway	*p*-value	Ecocyc pathway	*p*-value
Nitrate reduction	2.61×10^{-7}	Glycerol-3-phosphate to fumarate electron transfer	0.023
Respiration	2.61×10^{-7}	Superpathway of sulfate assimilation and cysteine biosynthesis	0.023
Nitrate reduction VIII (dissimilatory)	3.91×10^{-7}	L-arginine biosynthesis I (via L-ornithine)	0.023
Electron transfer chains	6.17×10^{-7}	L-arginine biosynthesis	0.023
Nitrogen compound metabolism	7.80×10^{-7}	Detoxification	0.023
NADH to cytochrome *bo* oxidase electron transfer I	2.43×10^{-6}	Assimilatory sulfate reduction	0.023
Anaerobic respiration	1.17×10^{-5}	Assimilatory sulfate reduction I	0.023
Nitrate reduction III (dissimilatory)	2.48×10^{-5}		
Nucleic acid processing	2.48×10^{-5}		
Aerobic respiration	1.62×10^{-4}		
Macromolecule modification	8.84×10^{-4}		
NADH to hydrogen peroxide electron transfer	9.19×10^{-4}		
Succinate to cytochrome *bo* oxidase electron transfer	2.43×10^{-3}		
L-histidine biosynthesis	2.43×10^{-3}		
ATP biosynthesis	2.43×10^{-3}		
L-histidine biosynthesis	2.43×10^{-3}		
NADH to trimethylamine *N*-oxide electron transfer	2.43×10^{-3}		
Inorganic nutrient metabolism	6.16×10^{-3}		
NADH to dimethyl sulfoxide electron transfer	6.54×10^{-3}		
Generation of precursor metabolites and energy	0.011		
Formate to nitrite electron transfer	0.013		
NADH to cytochrome *bd* oxidase electron transfer I	0.014		
NADH to fumarate electron transfer	0.014		

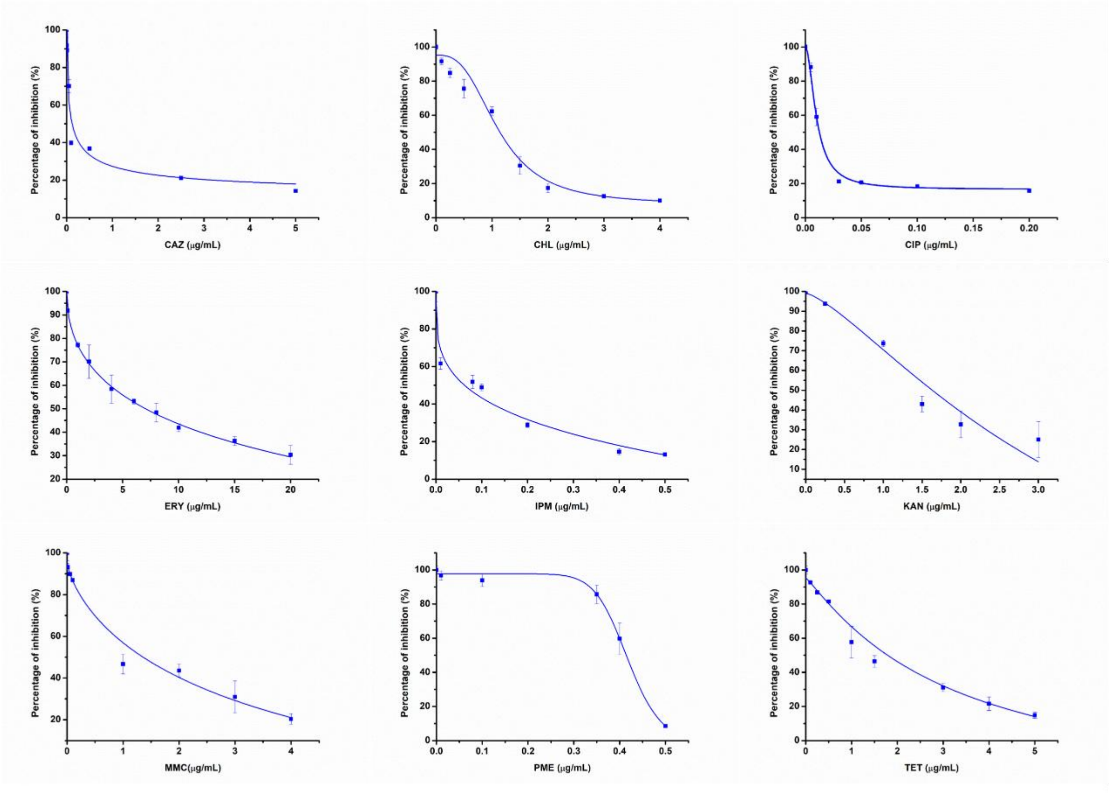

Figure S1. Response of *E. coli* growth to antibiotics. TET, Tetracycline; Mitomycin C, MMC; Imipenem, IPM; Ceftazidime, CAZ; Kanamycin, KAN; Ciprofloxacin, CIP; Polymyxin E, PME; Chloramphenicol, CHL; Erythromycin, ERY. Error bar indicates standard deviation of three replicates.

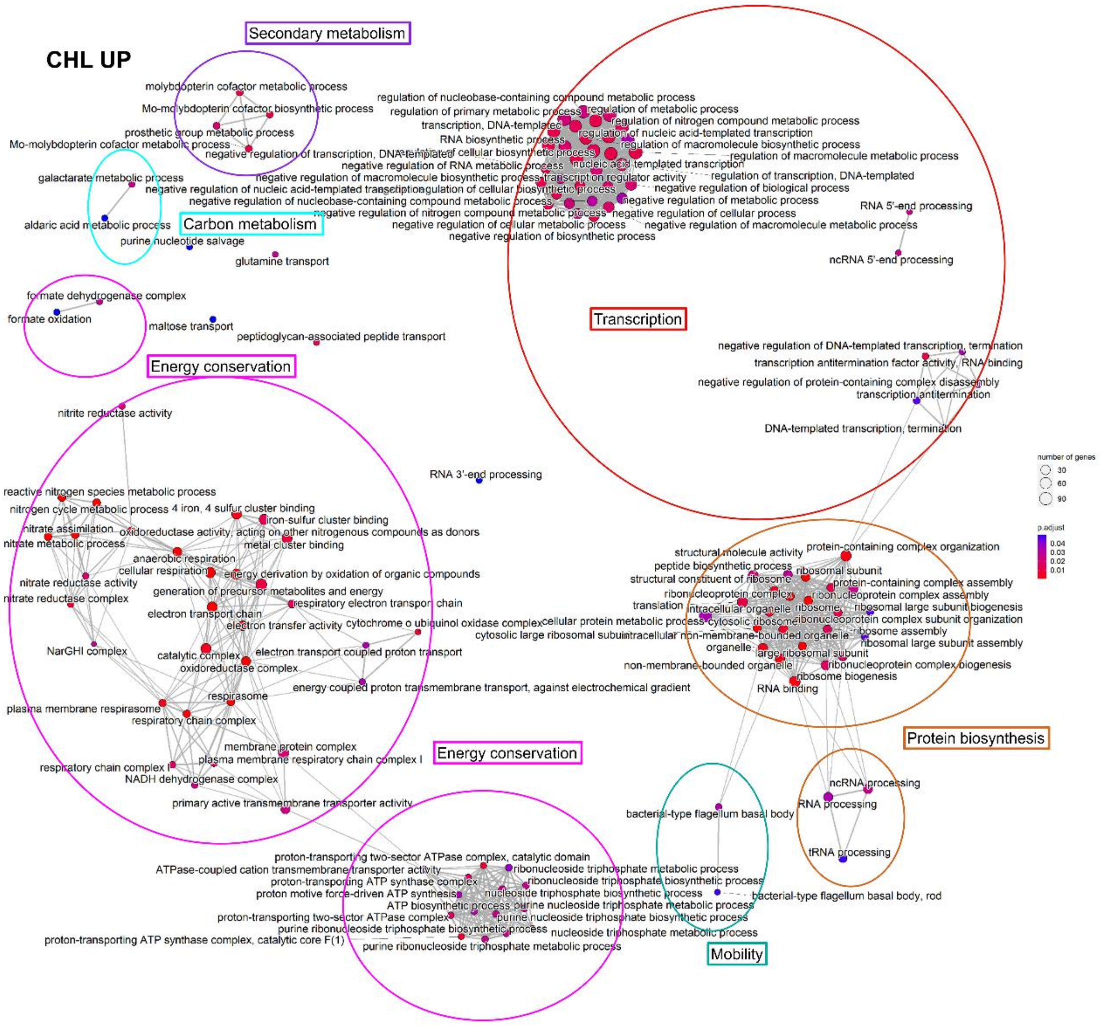

Figure S2. Upregulated gene ontology terms upon treatment with chloramphenicol determined with enrichment analysis.

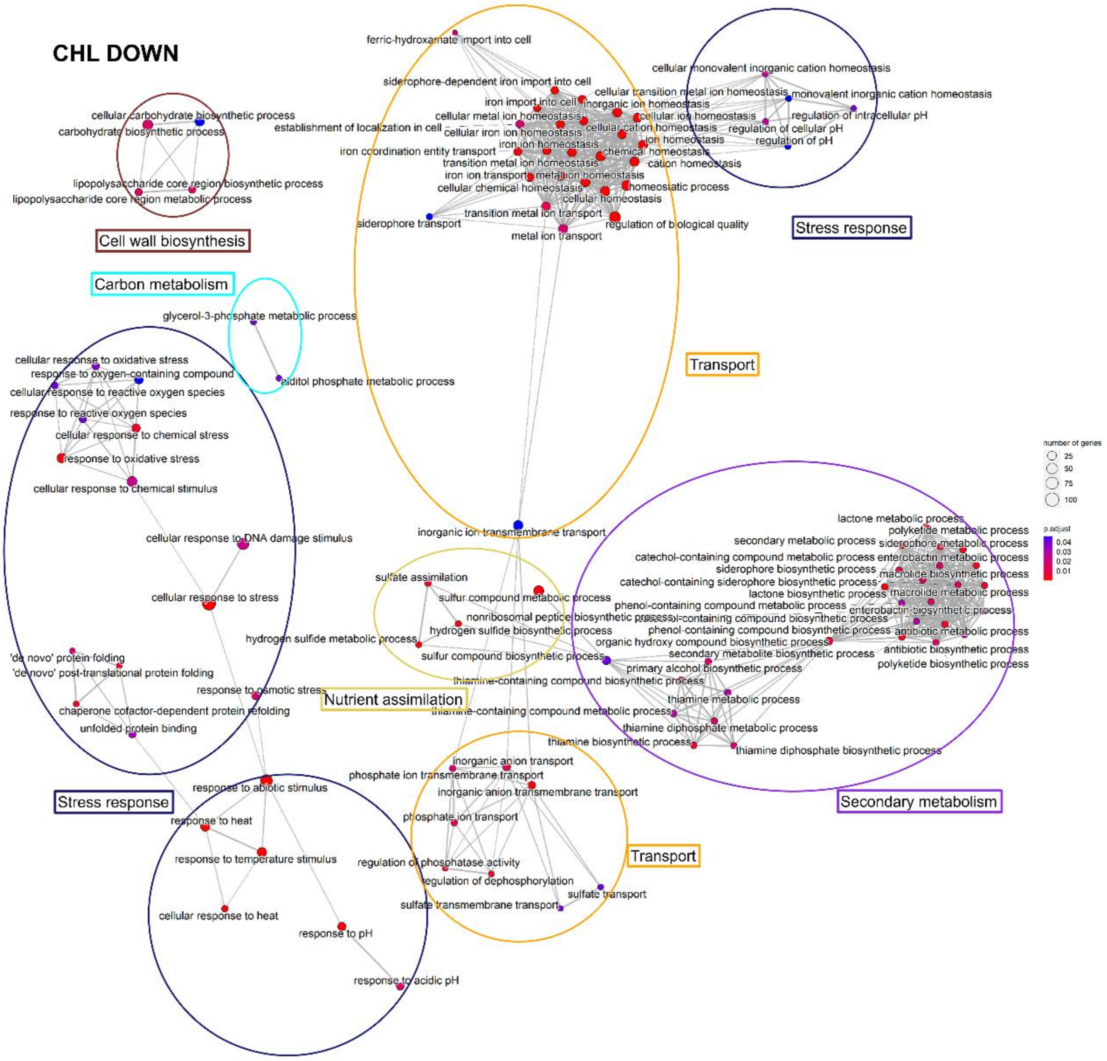

Figure S3. Downregulated gene ontology terms upon treatment with chloramphenicol determined with enrichment analysis.

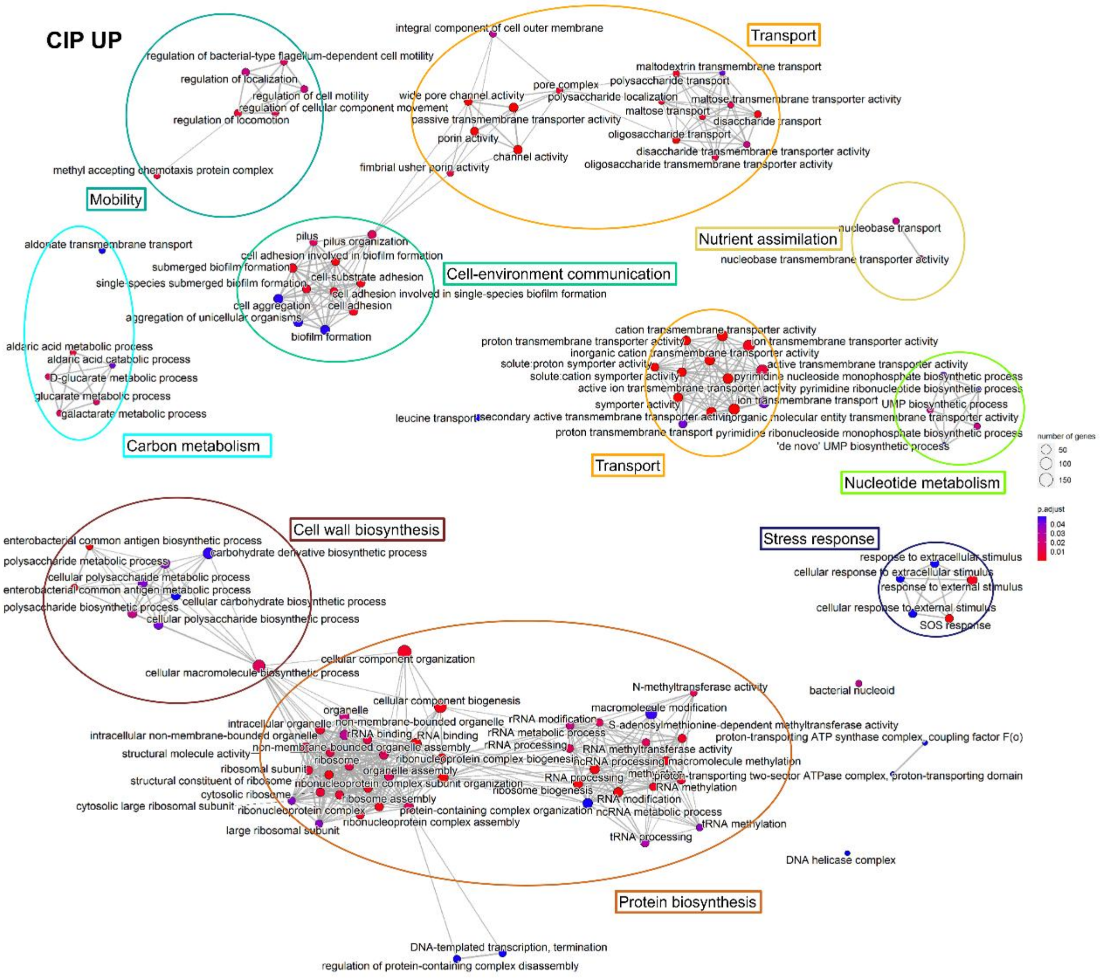

Figure S4. Upregulated gene ontology terms upon treatment with ciprofloxacin determined with enrichment analysis.

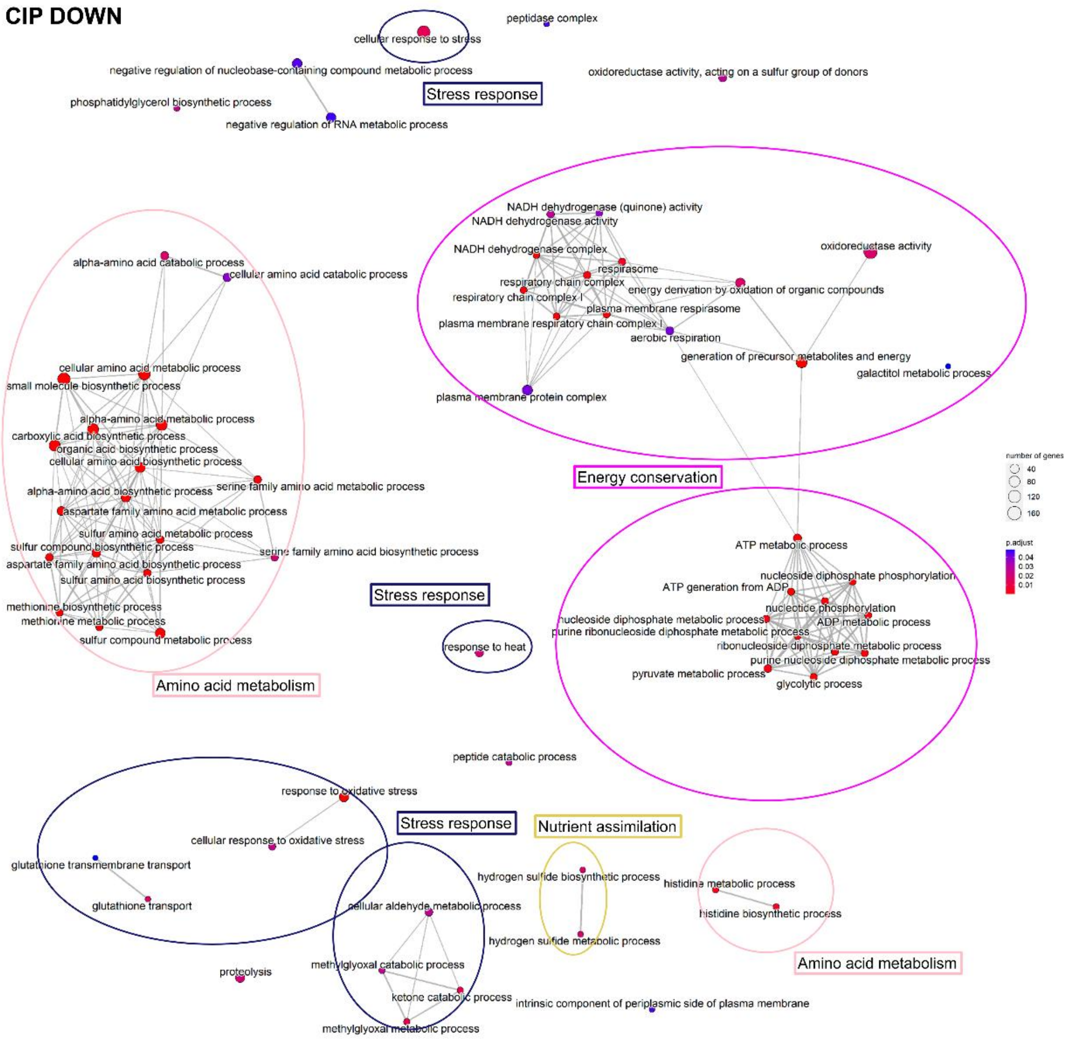

Figure S5. Downregulated gene ontology terms upon treatment with ciprofloxacin determined with enrichment analysis.

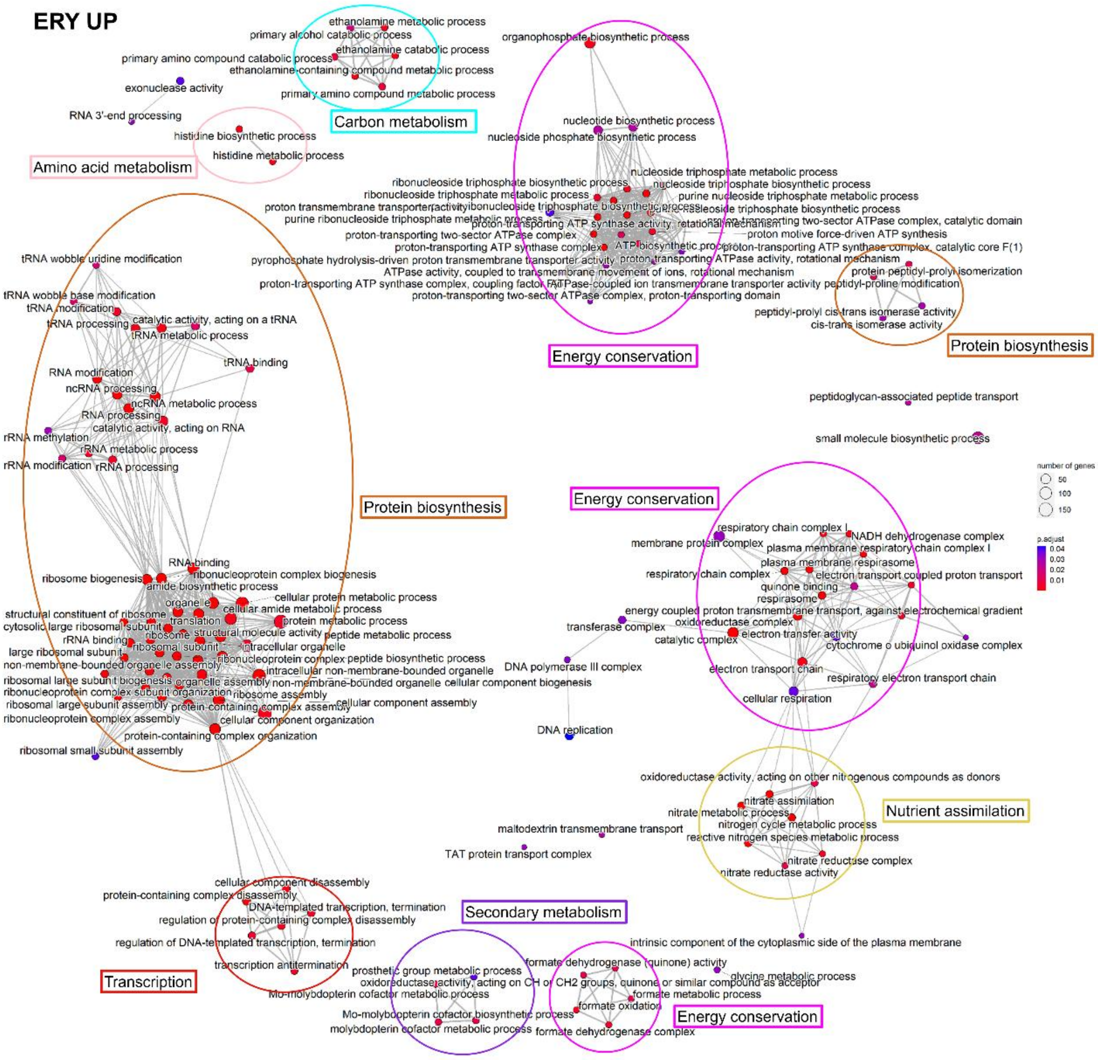

Figure S6. Upregulated gene ontology terms upon treatment with erythromycin determined with enrichment analysis.

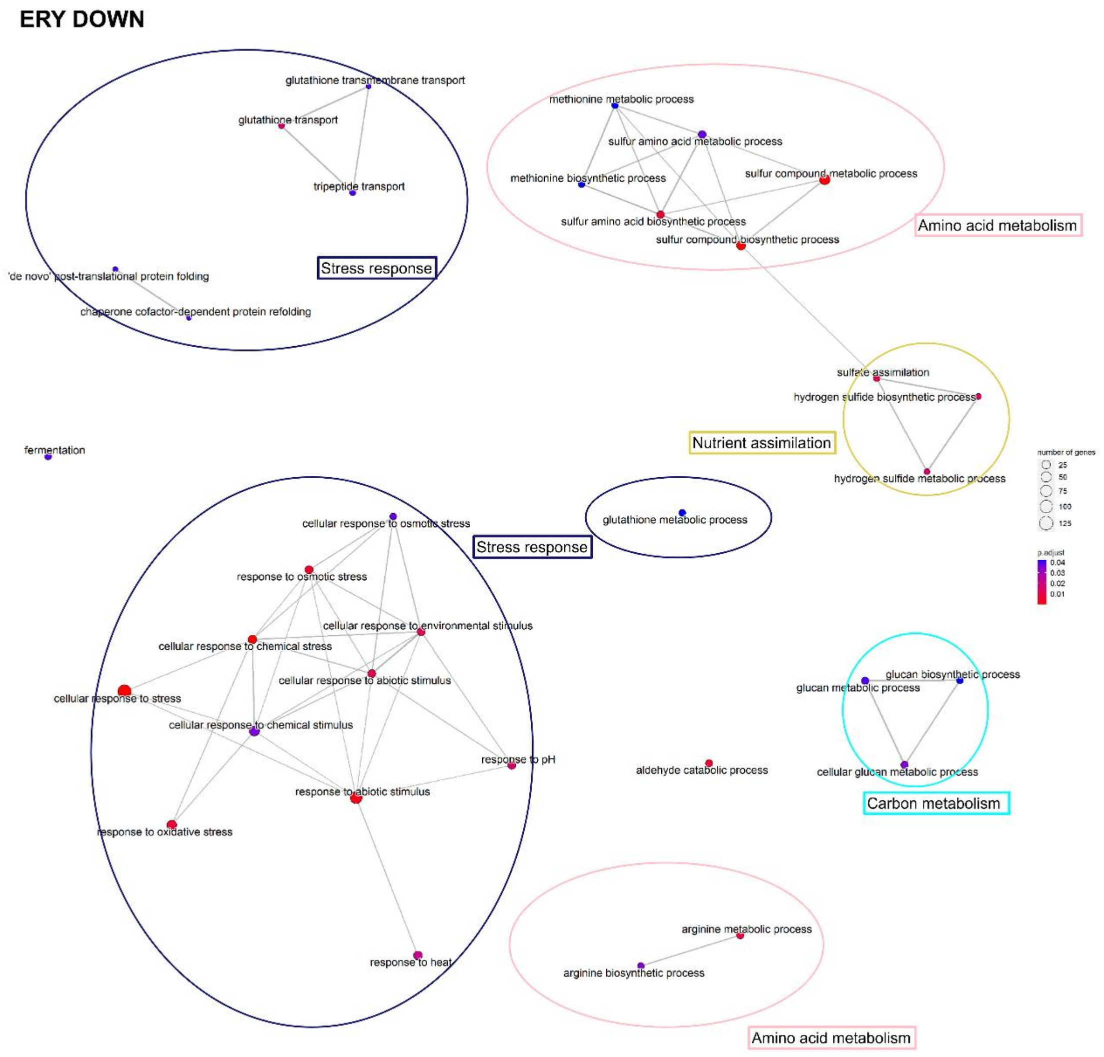

Figure S7. Downregulated gene ontology terms upon treatment with erythromycin determined with enrichment analysis.

TET UP

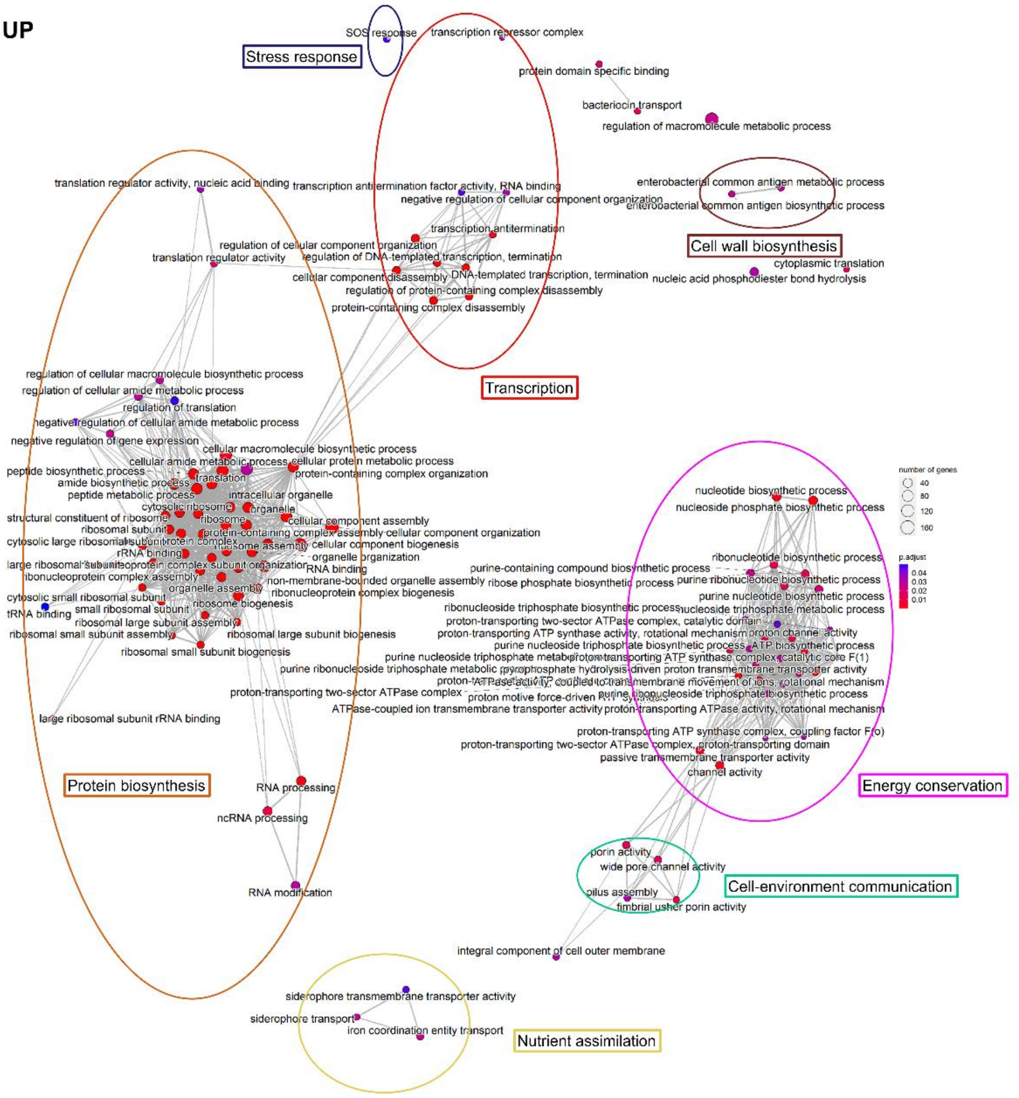

Figure S8. Upregulated gene ontology terms upon treatment with tetracycline determined with enrichment analysis.

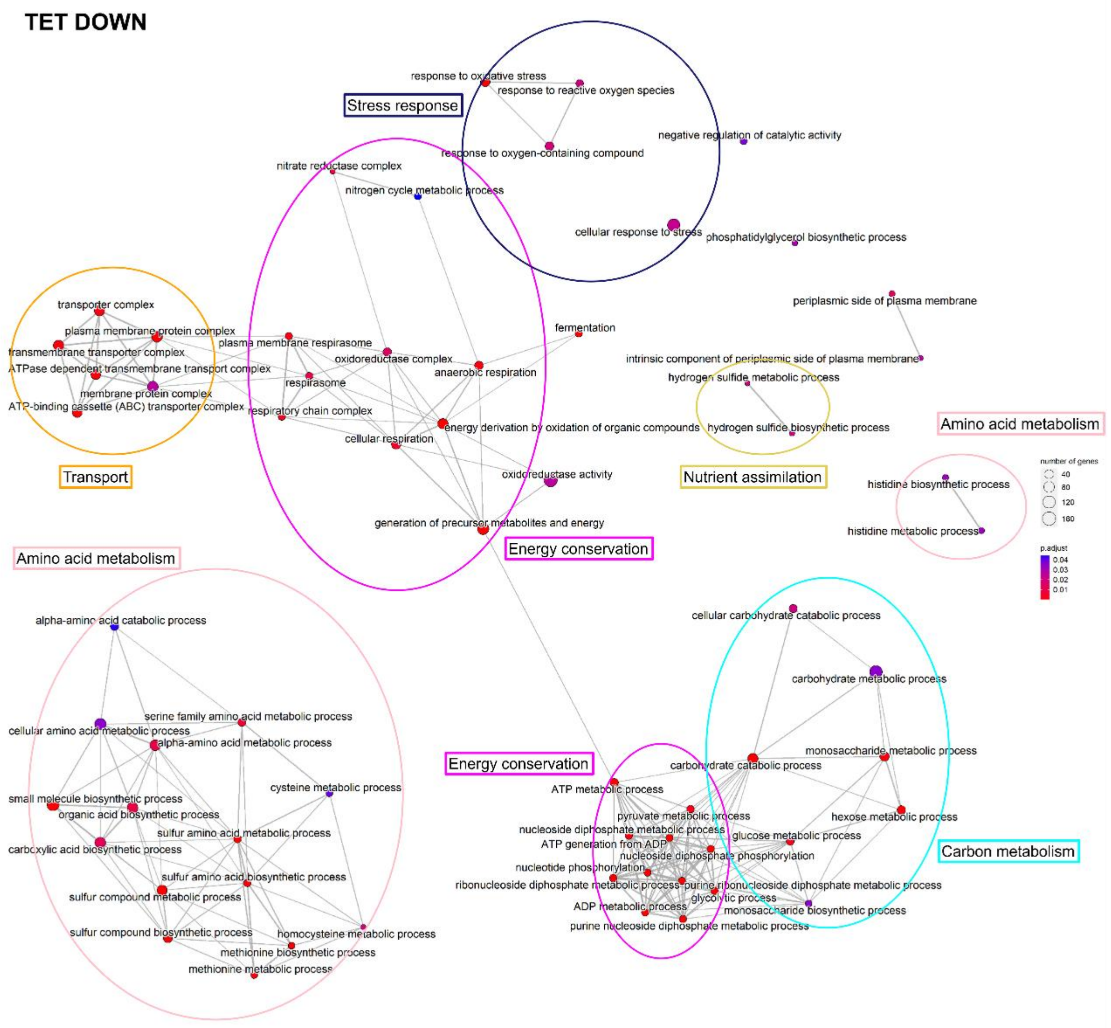

Figure S9. Downregulated gene ontology terms upon treatment with tetracycline determined with enrichment analysis.

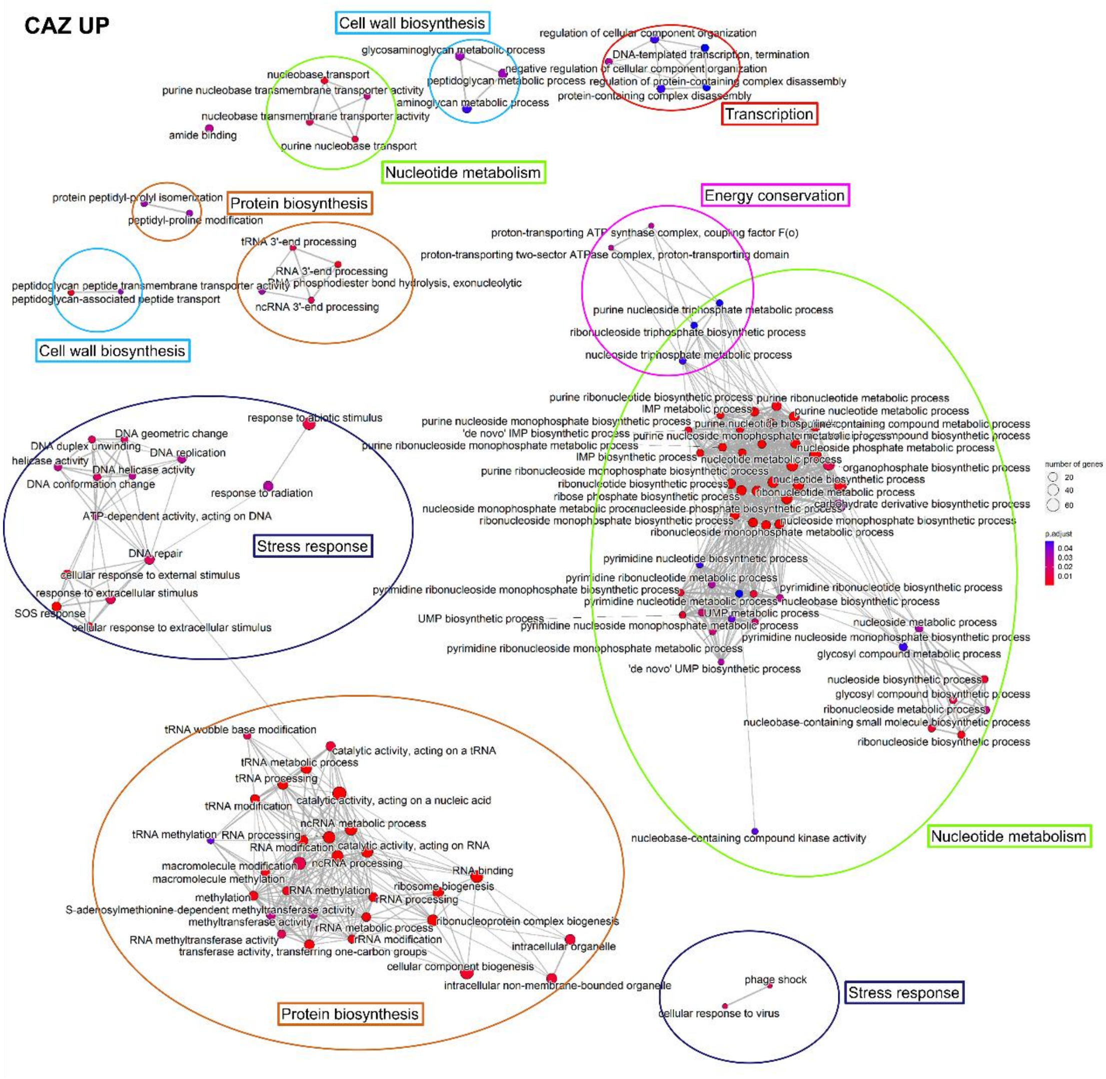

Figure S10. Upregulated gene ontology terms upon treatment with ceftazidime determined with enrichment analysis.

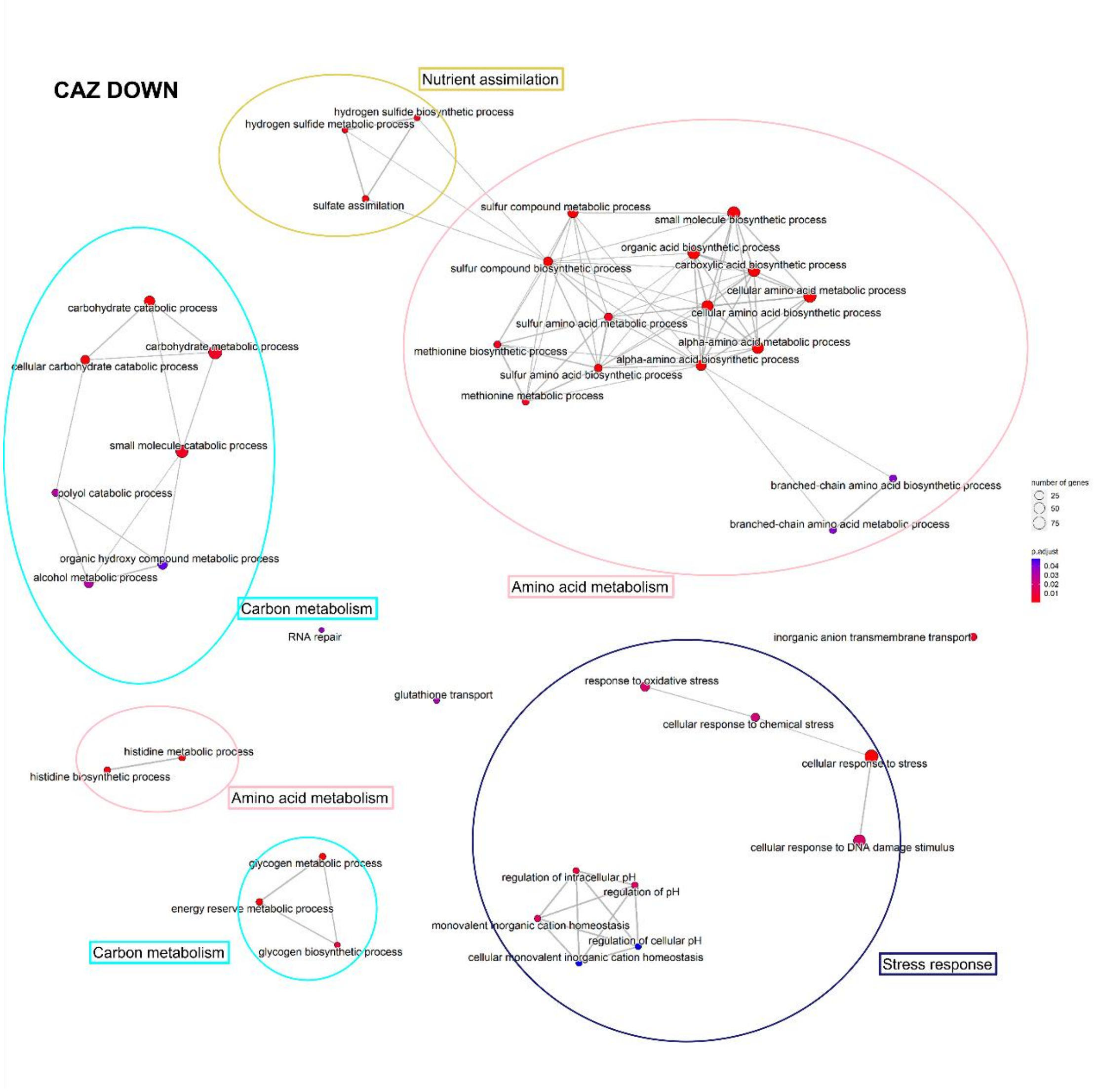

Figure S11. Downregulated gene ontology terms upon treatment with ceftazidime determined with enrichment analysis.

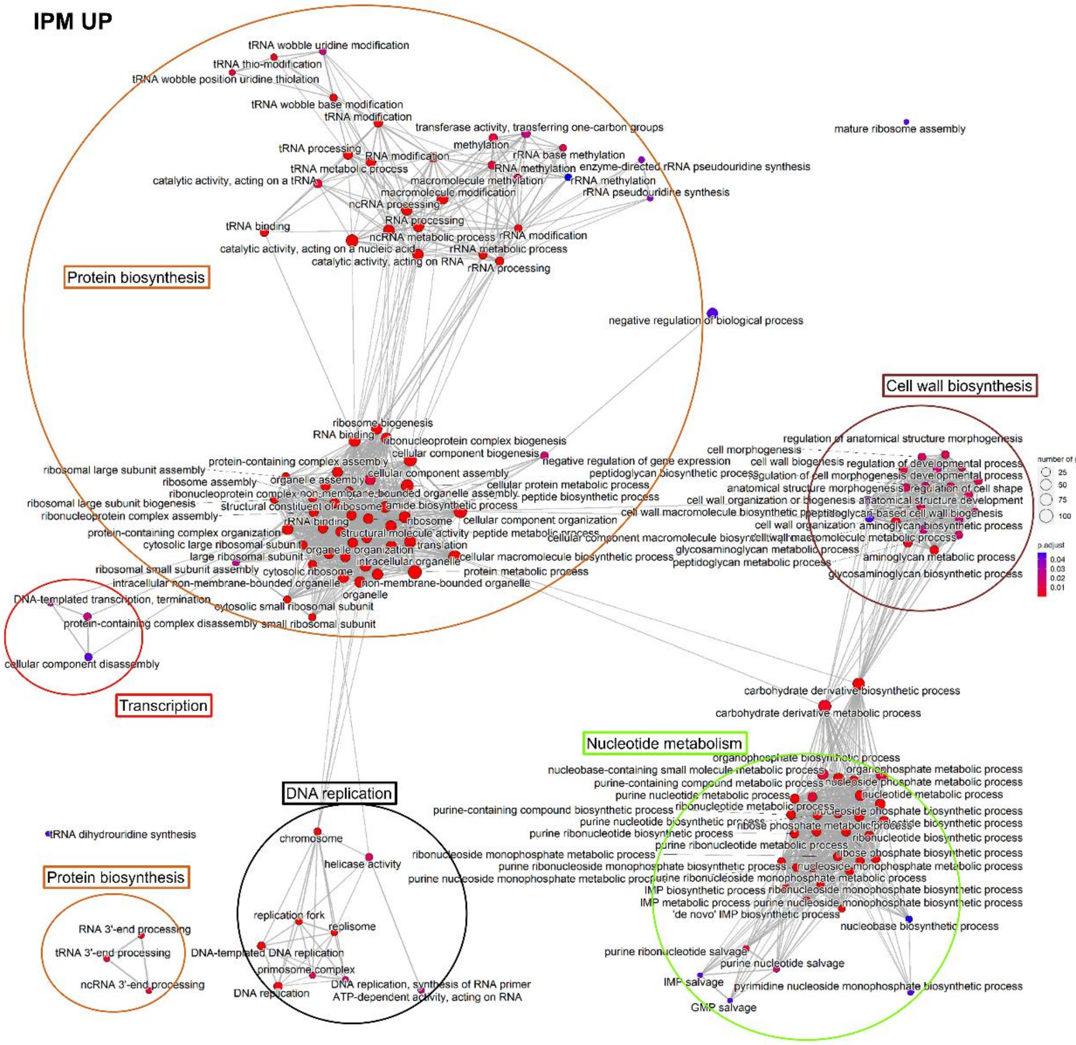

Figure S12. Upregulated gene ontology terms upon treatment with imipenem determined with enrichment analysis.

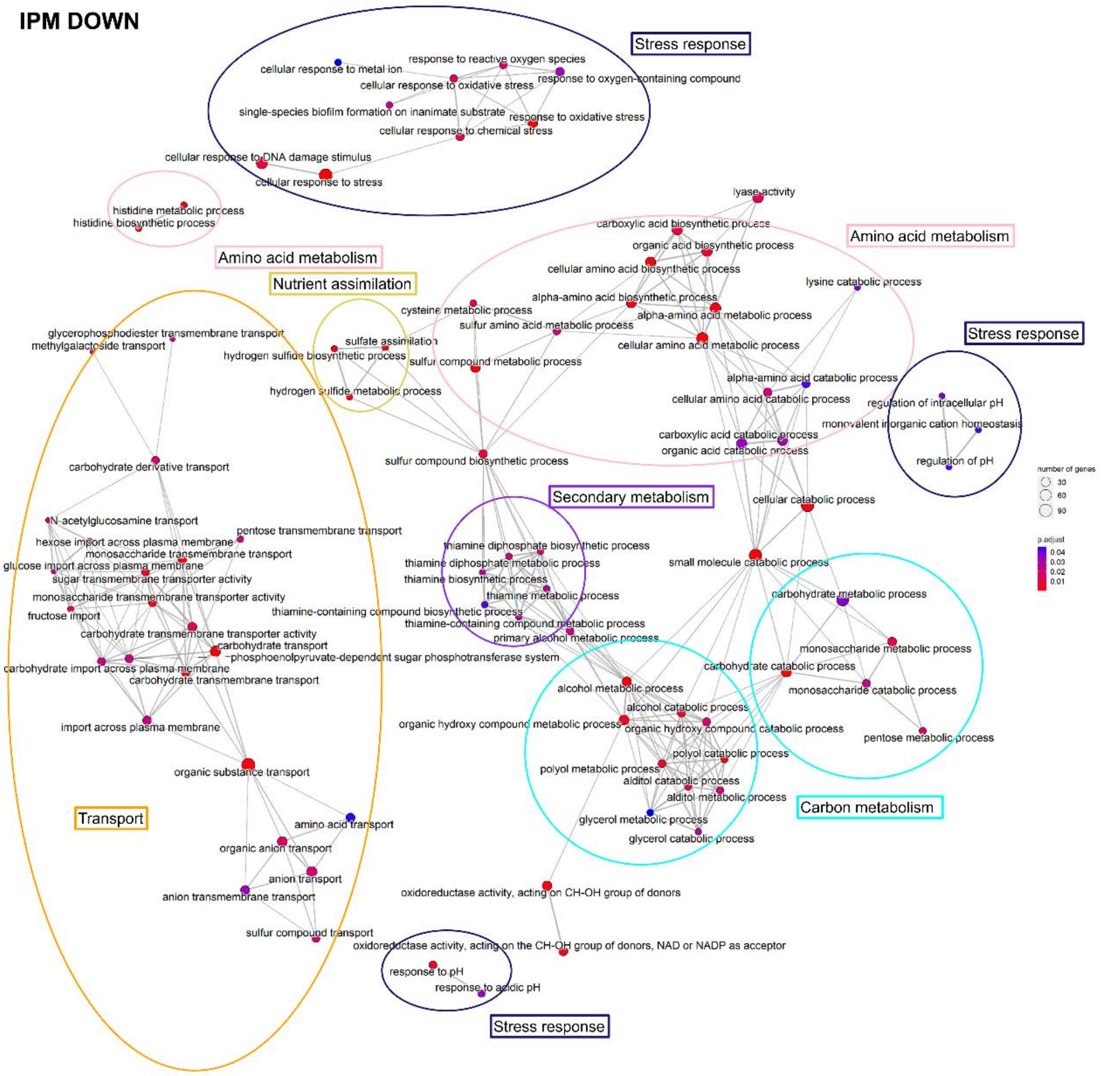

Figure S13. Downregulated gene ontology terms upon treatment with imipenem determined with enrichment analysis.

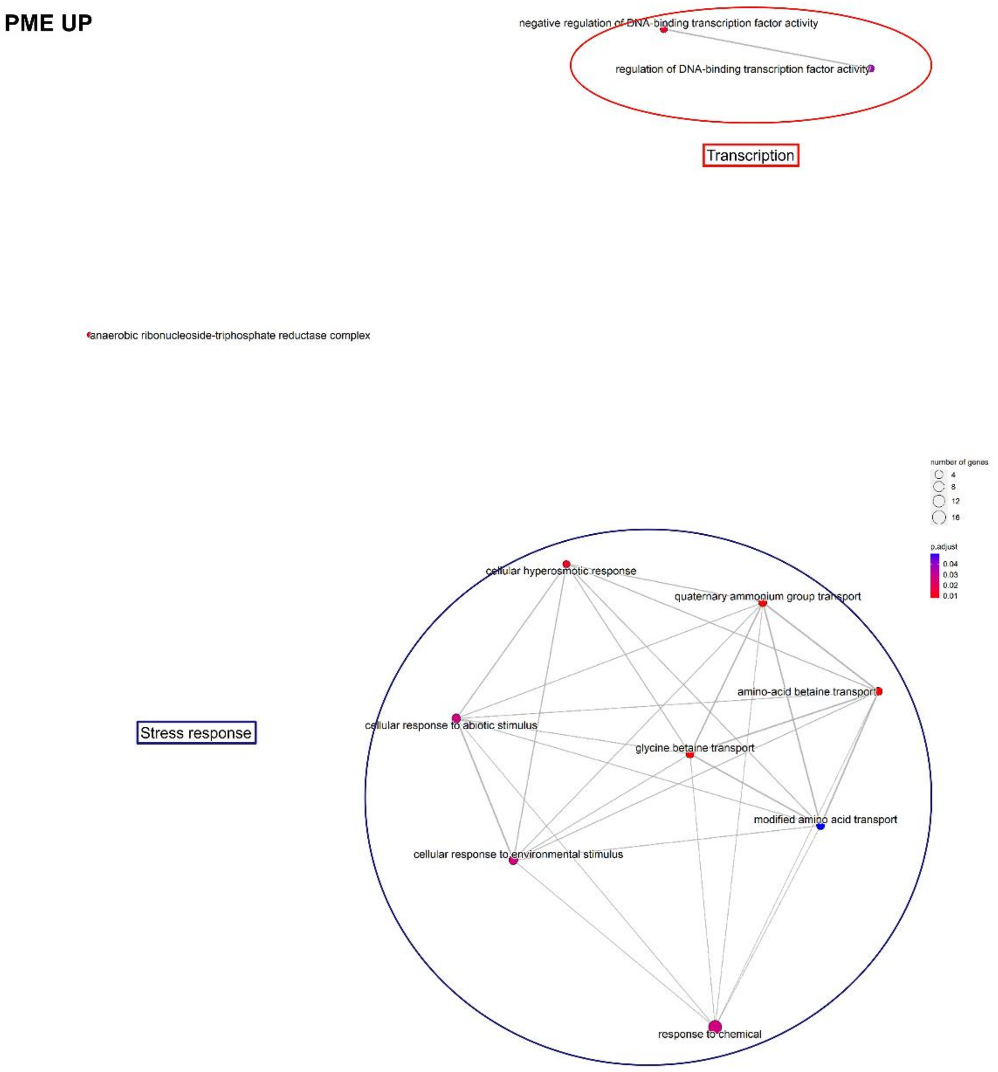

Figure S14. Upregulated gene ontology terms upon treatment with polymyxin E determined with enrichment analysis.

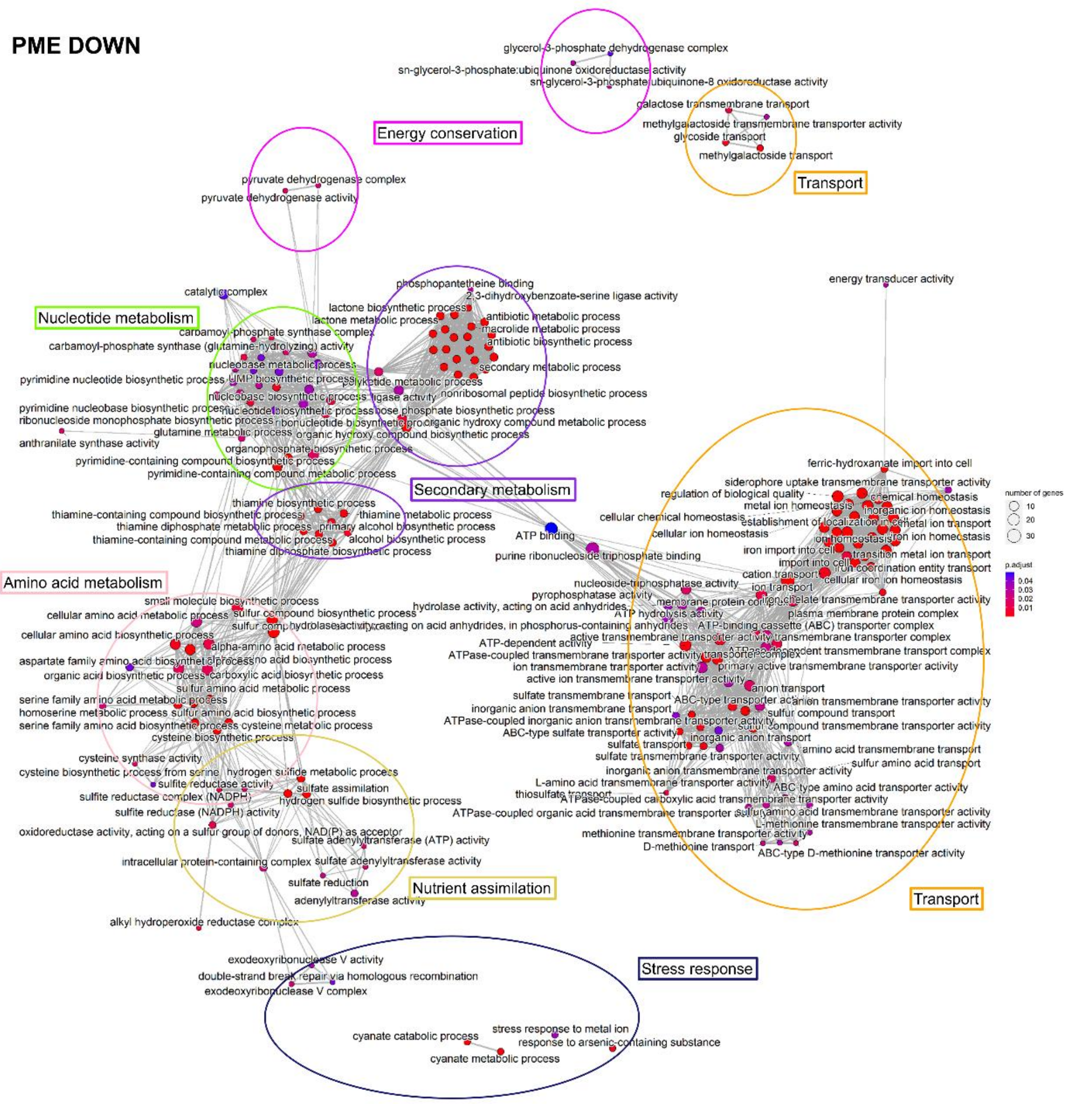

Figure S15. Downregulated gene ontology terms upon treatment with polymyxin E determined with enrichment analysis.

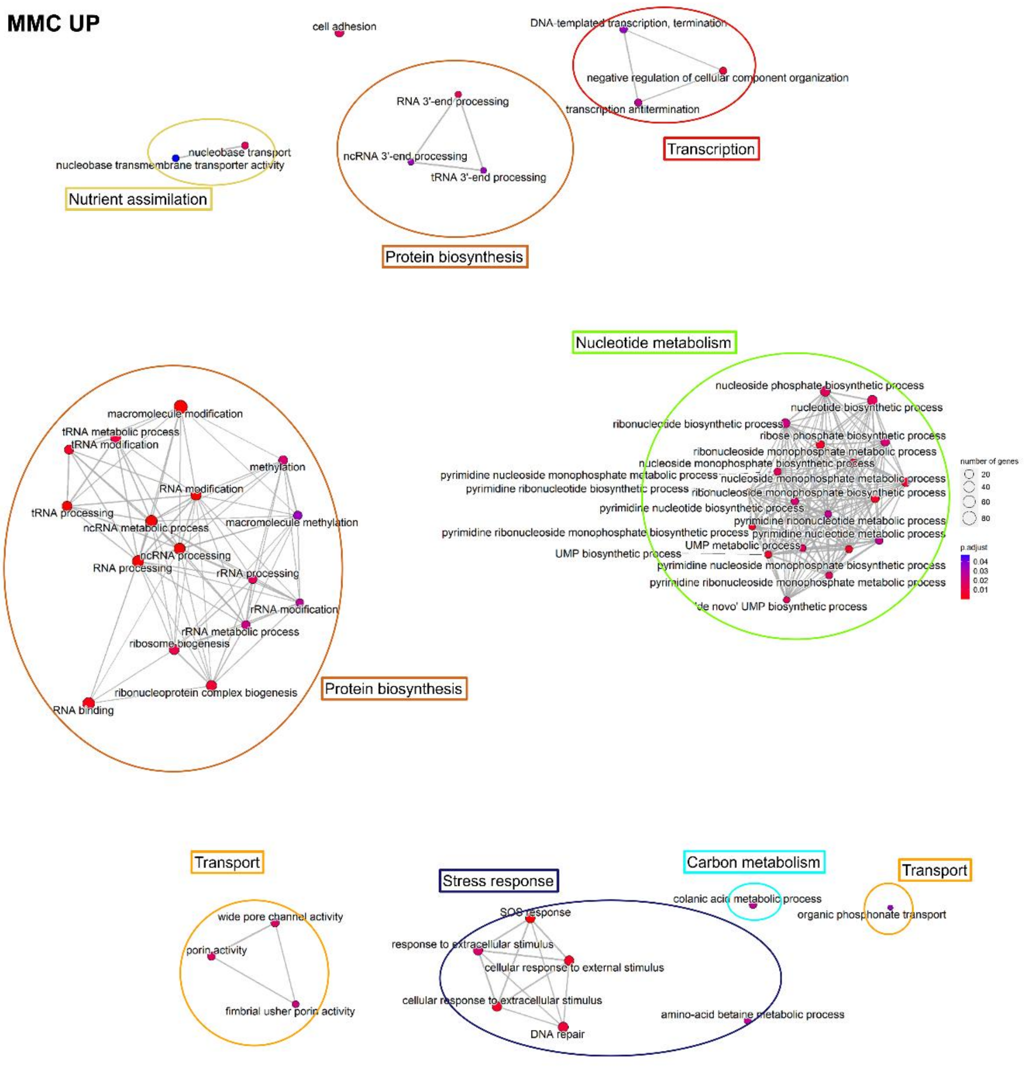

Figure S16. Upregulated gene ontology terms upon treatment with mitomycin C determined with enrichment analysis.

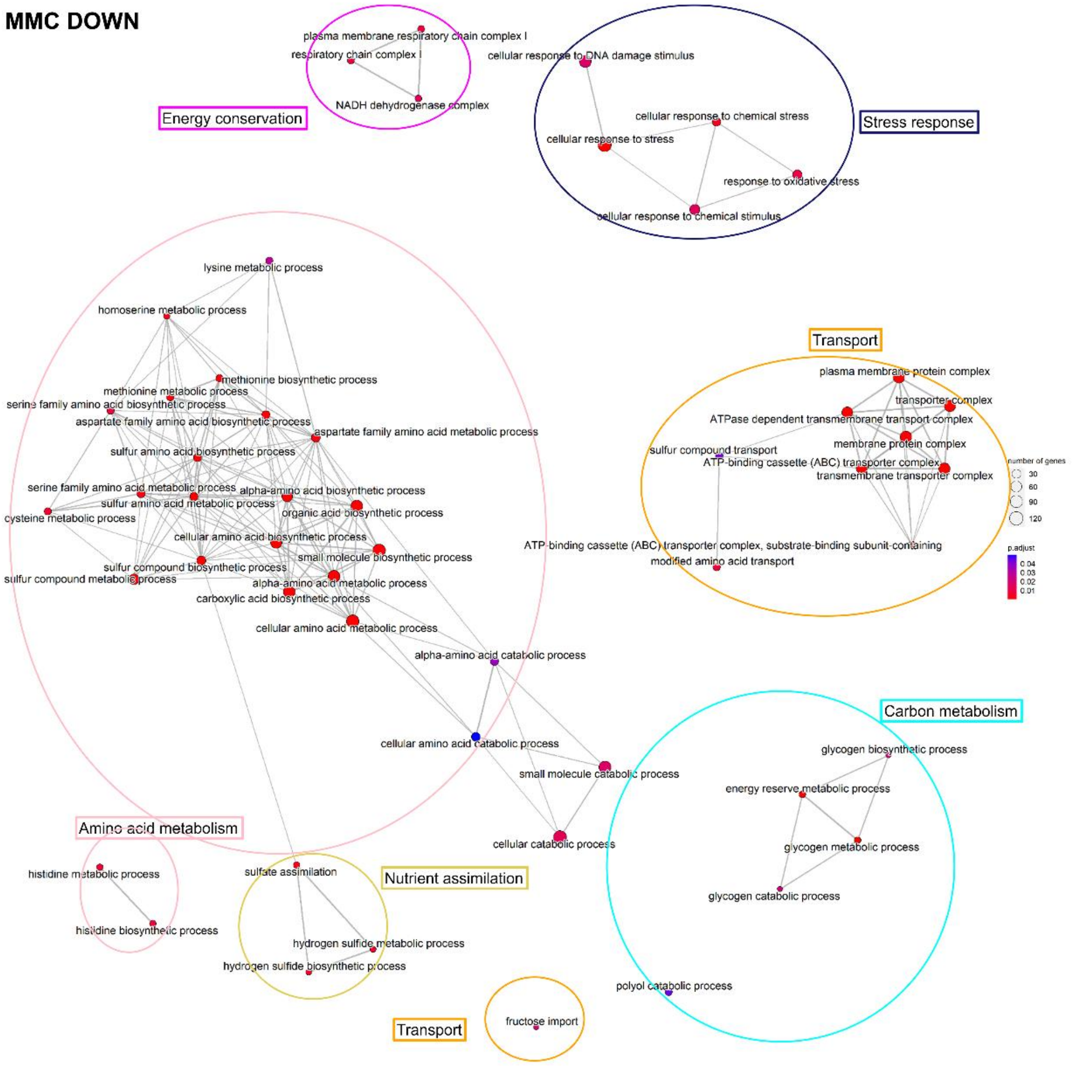

Figure S17. Downregulated gene ontology terms upon treatment with mitomycin C determined with enrichment analysis.

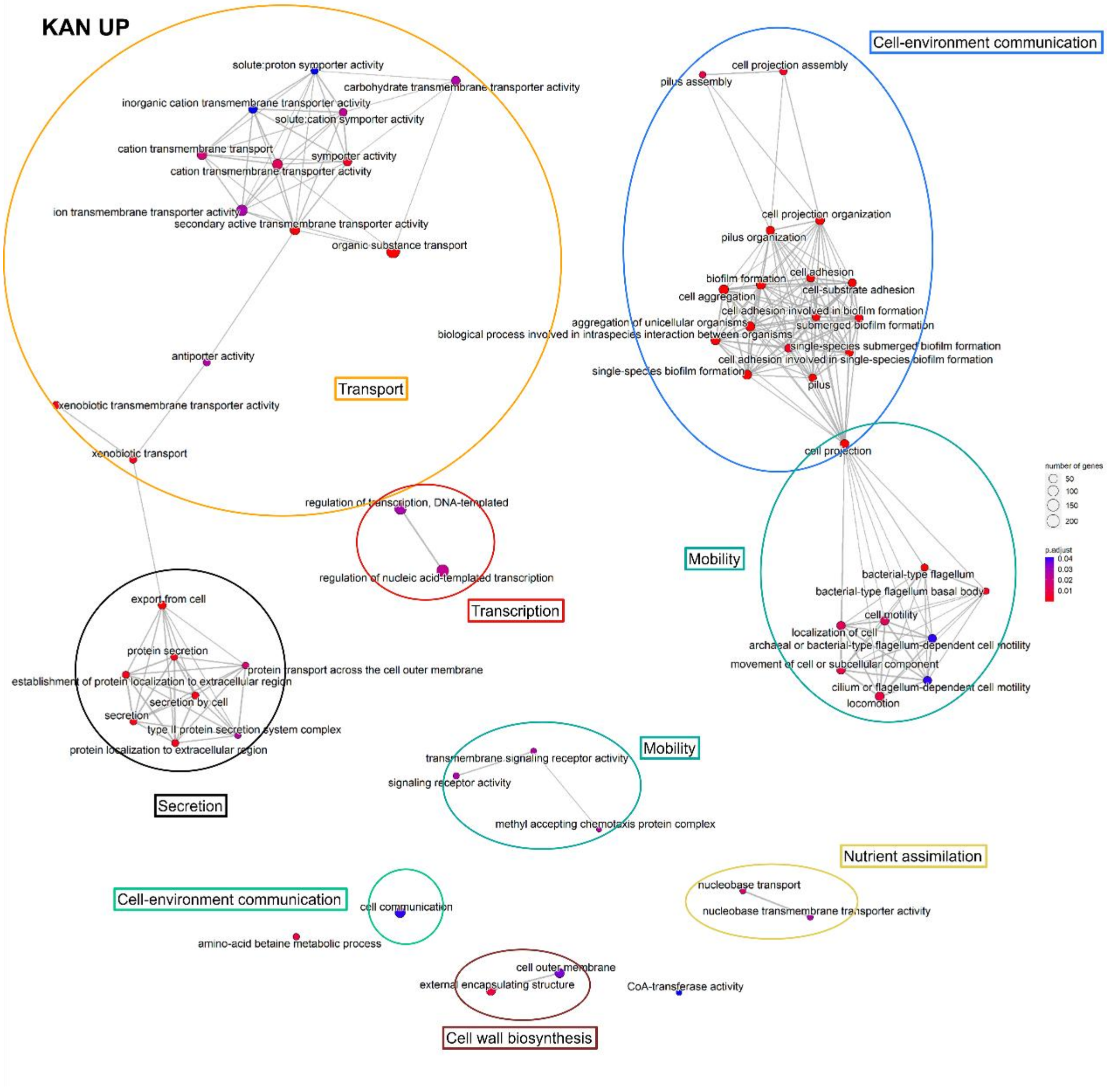

Figure S18. Upregulated gene ontology terms upon treatment with kanamycin determined with enrichment analysis.

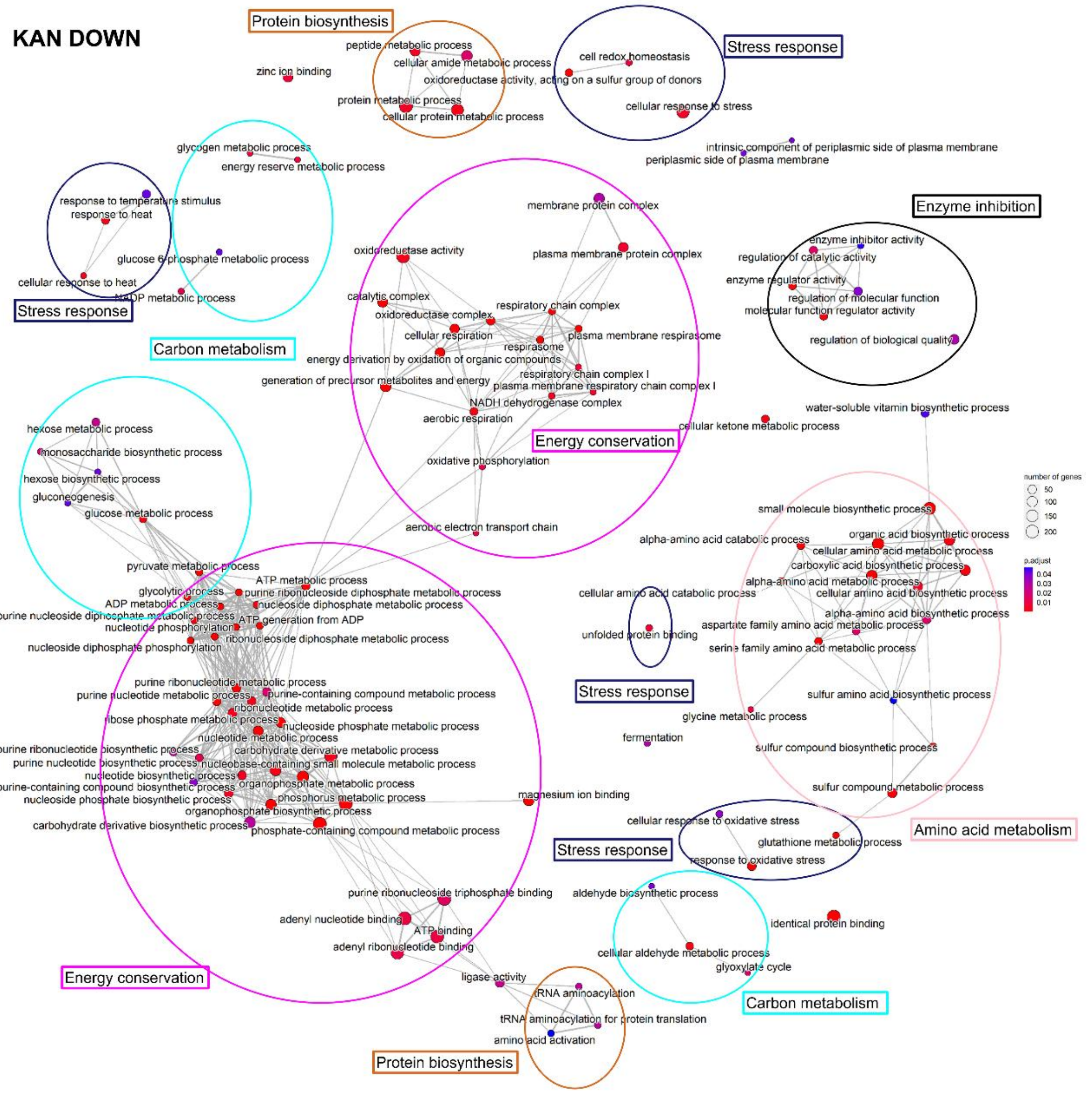

Figure S19. Downregulated gene ontology terms upon treatment with kanamycin determined with enrichment analysis.

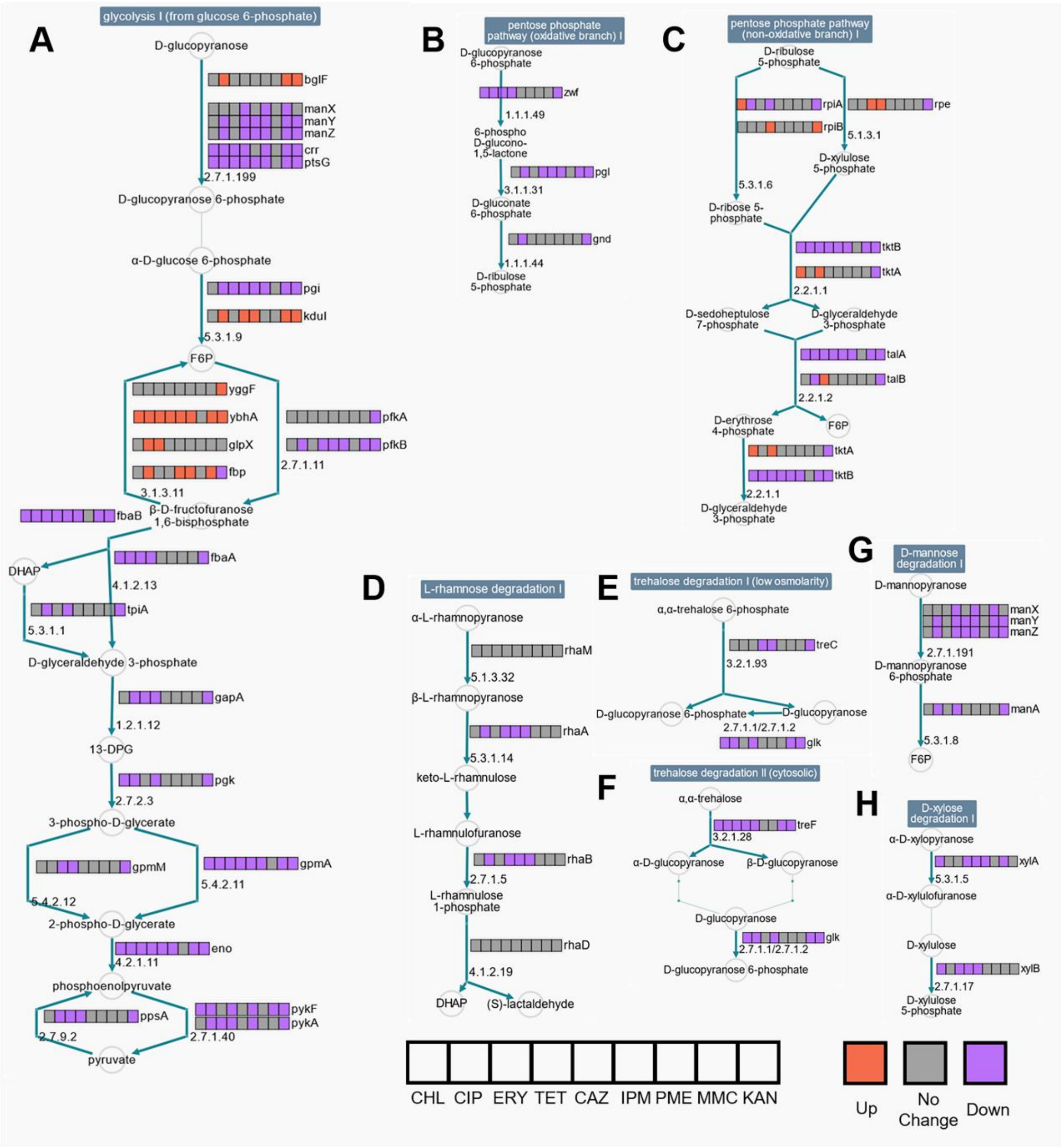

Figure S20. Response of carbohydrate metabolism to antibiotics. Panel A, glycolysis pathway; Panel B, pentose phosphate pathway (oxidative branch); Panel C, pentose phosphate pathway (non-oxidative branch); Panel D, rhamnose metabolism; Panel E, trehalose metabolism (low osmolarity); Panel F, trehalose metabolism (cytosolic); Panel G, mannose metabolism; Panel H, xylose metabolism. EC numbers are indicated beside genes.

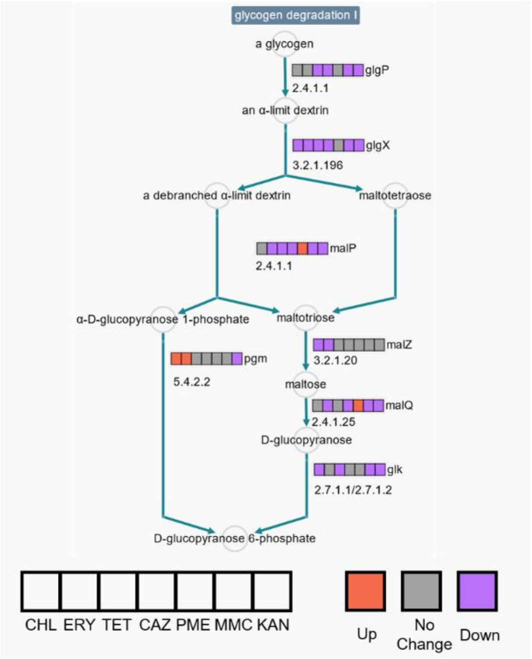

Figure S21. Response of glycogen metabolism to antibiotics.

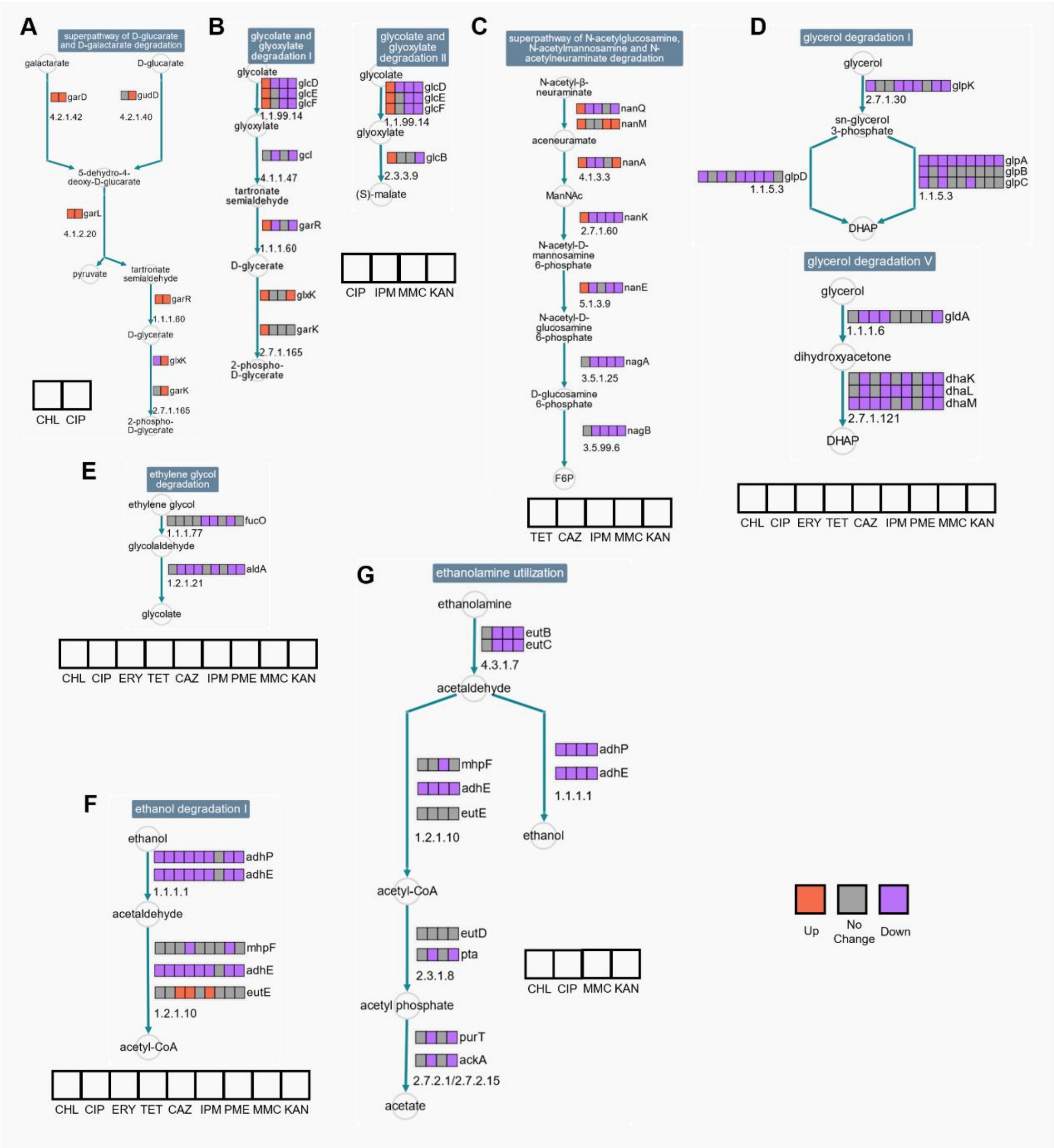

Figure S22. Response of non-carbohydrate carbon metabolism to antibiotics.

Panel A, aldaric acid metabolism; Panel B, glycolate and glyoxylate metabolism; Panel C, amino sugar metabolism; Panel D, glycerol metabolism; Panel E, ethylene glycol metabolism; Panel F, ethanol metabolism; Panel G, ethanolamine metabolism. F6P, β-D-fructofuranose 6-phosphate; DHAP, glycerone phosphate. EC numbers are indicated beside genes.

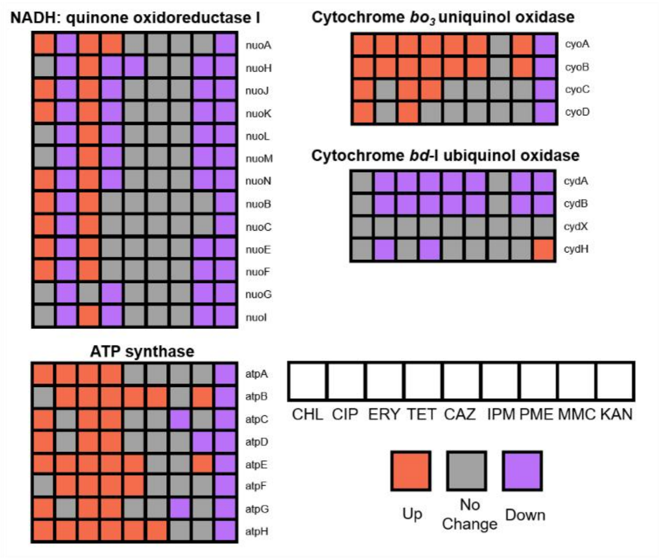

Figure S23. Transcriptomic response of energy conservation.

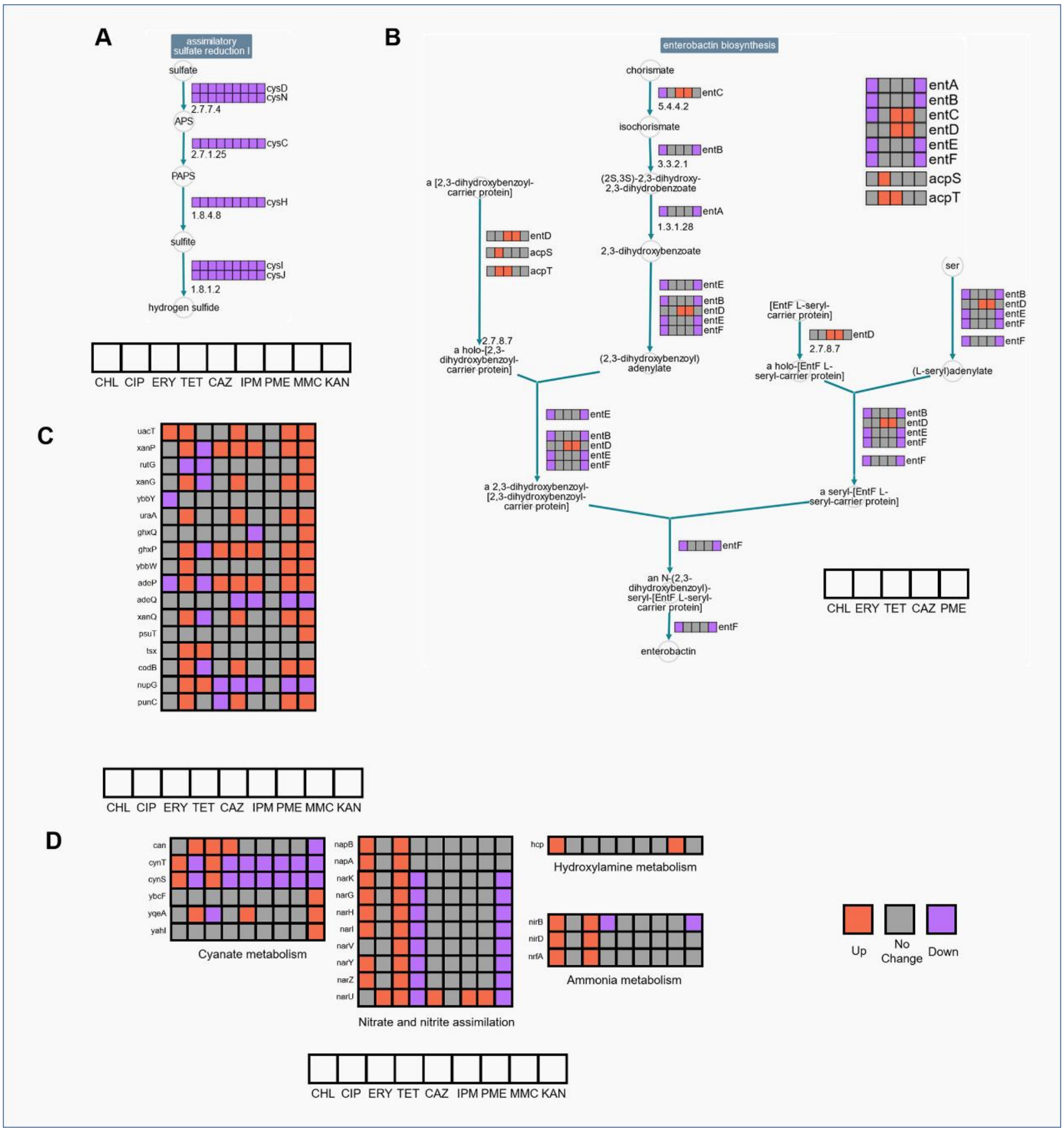

Figure S24. Response of nutrient assimilation pathways to antibiotics. Panel A, sulfate assimilation pathway; Panel B, iron assimilation pathway; Panel C, nucleobase transport pathway; Panel D, nitrogen metabolism pathway. EC numbers are indicated beside genes.

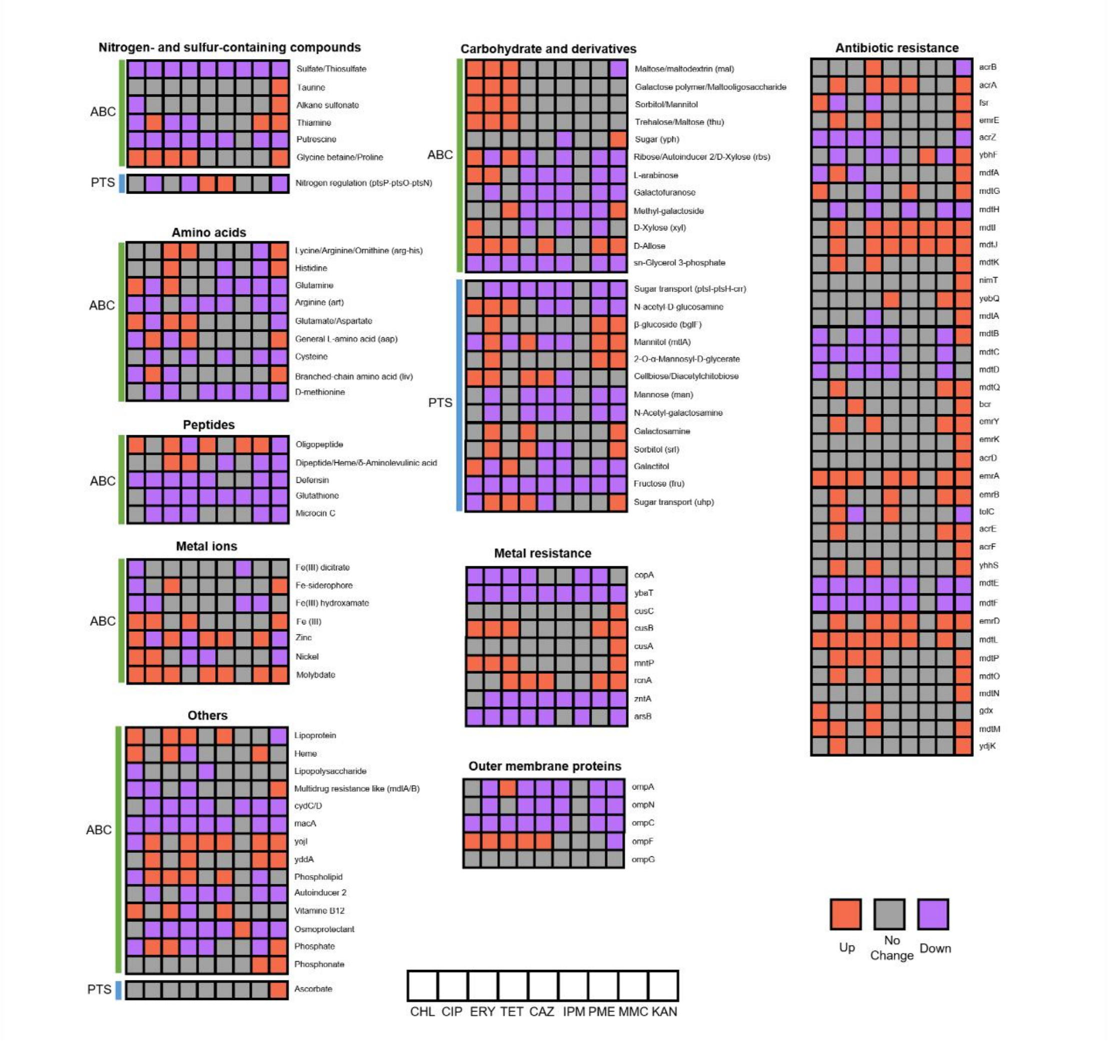

Figure S25. Response of transporter-coding genes to antibiotics.

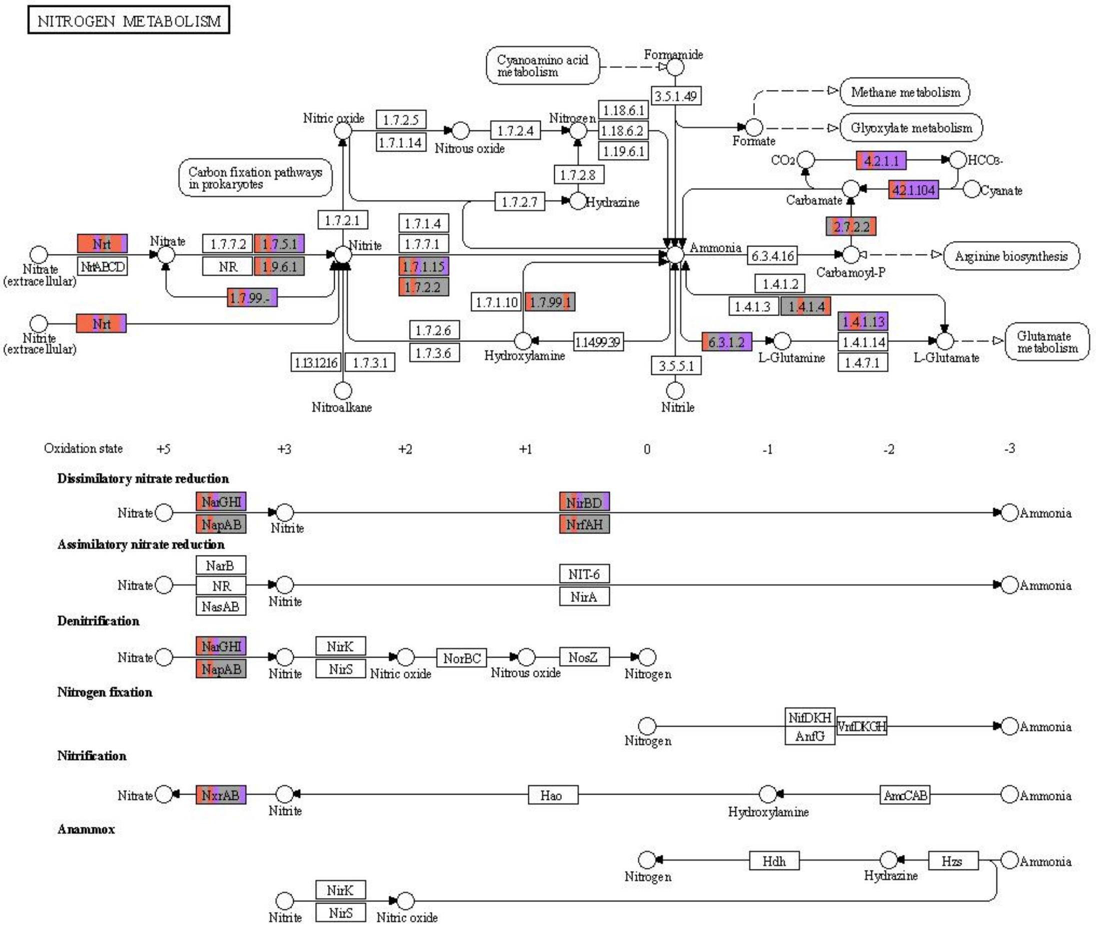

Figure S26. Regulation of nitrogen metabolism pathways. In each gene-representing square, colors indicate the regulation of the gene’s expression. From left to right in each square the colors represent regulation of chloramphenicol, ciprofloxacin, erythromycin, tetracycline, ceftazidime, imipenem, polymyxin E, mitomycin C, and kanamycin. Red color indicates upregulation. Purple color indicates downregulation.

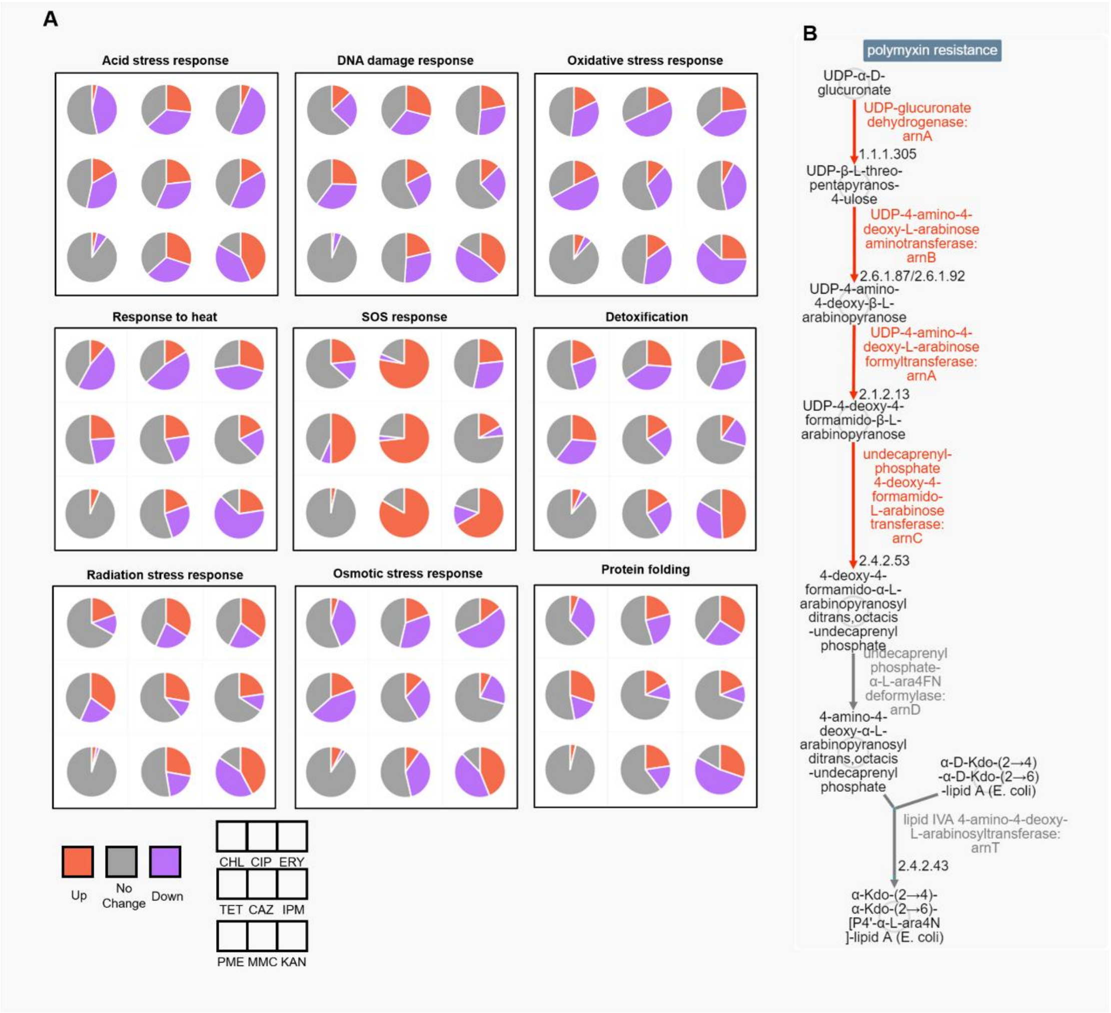

Figure S27. Transcriptomic response in stress response pathways. Panel A, stress response pathways, circles indicate proportions of upregulated, downregulated and unregulated genes; Panel B, polymyxin resistance pathway response to PME. EC numbers are indicated beside genes.

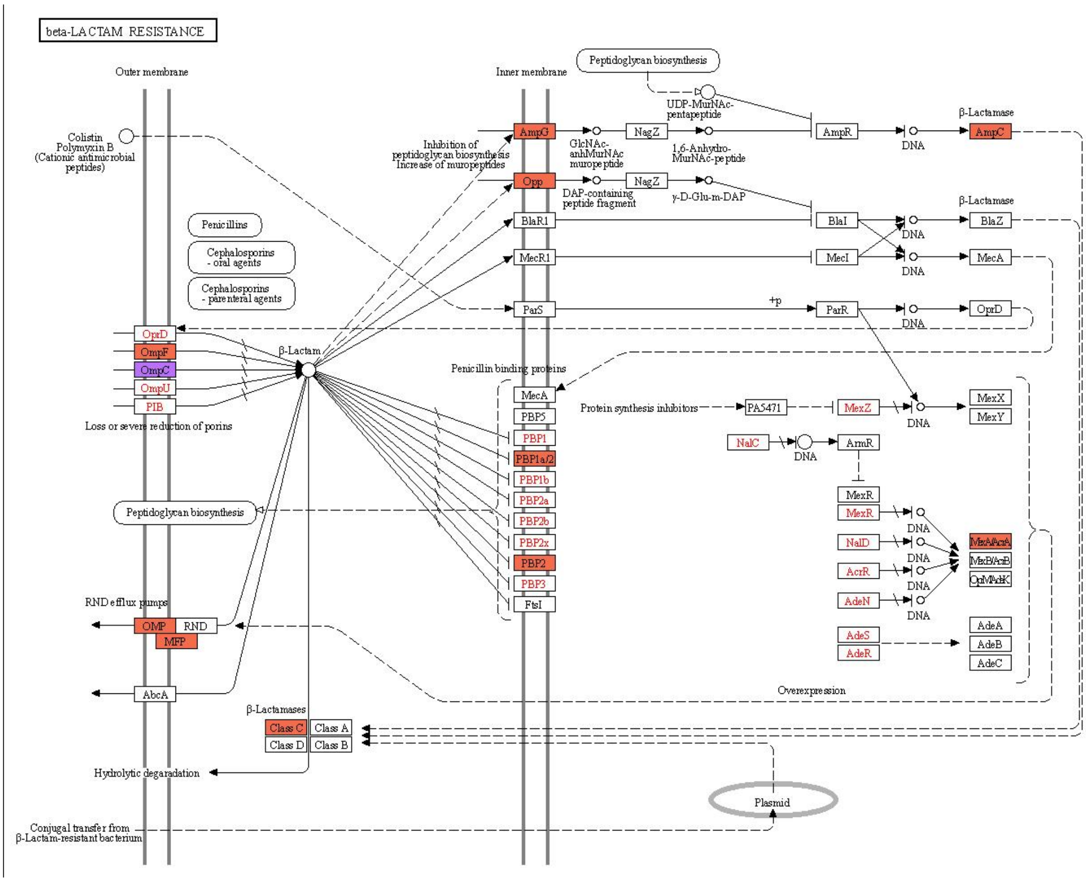

Figure S28. Transcriptomic response of β-lactam resistance pathway to CAZ.

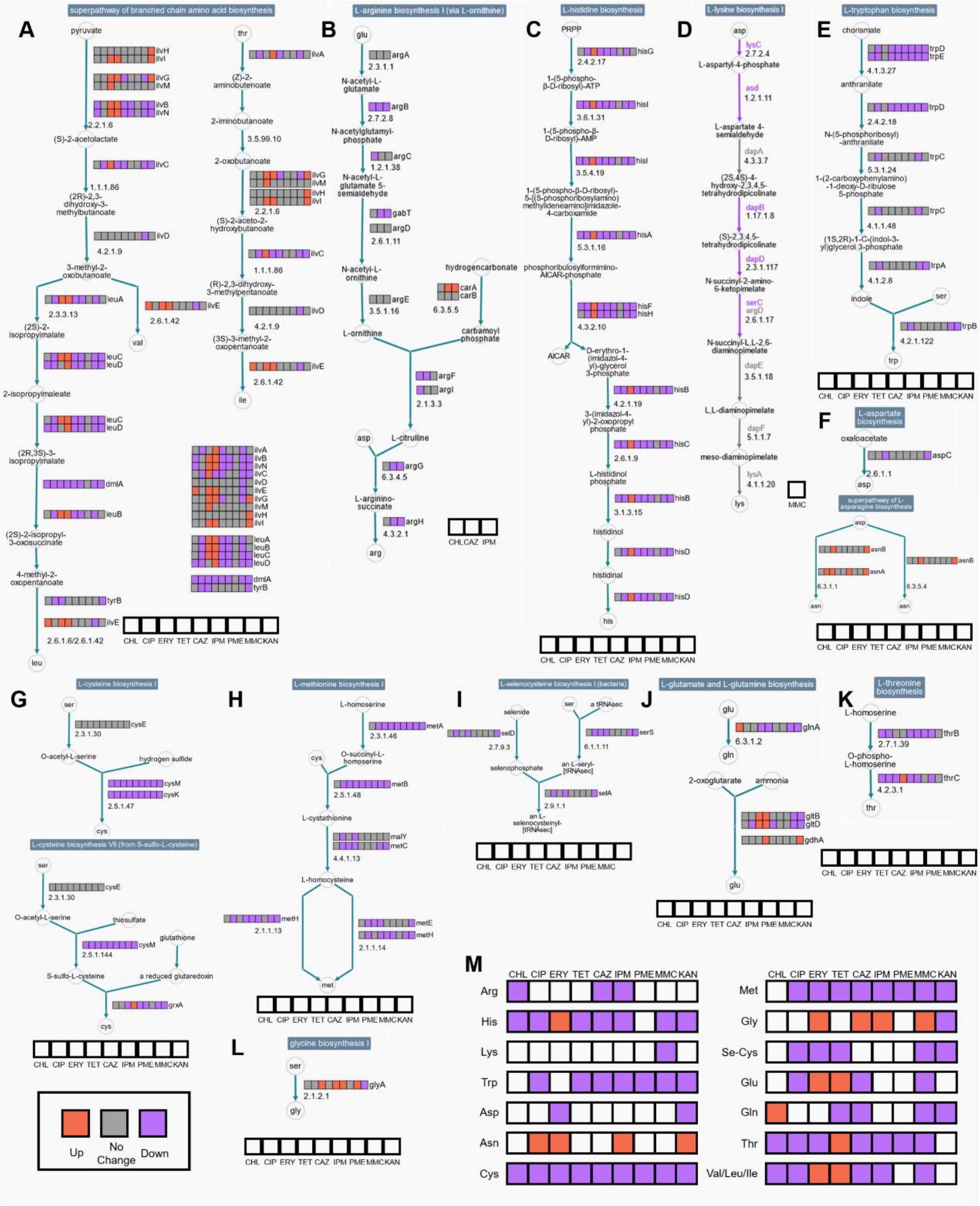

Figure S29. Response of amino acid metabolic pathways to antibiotics. Panel A, metabolism of valine, leucine, and isoleucine; Panel B, arginine metabolism pathway; panel C, histidine metabolism pathway; Panel D, lysine metabolism pathway; Panel E, tryptophan metabolism pathway; Panel F, asparagine and aspartic acid metabolism pathway; Panel G, cystine metabolism pathway; Panel H, methionine metabolism Panel I, selenocysteine metabolism pathway; Panel J, glutamine and glutamic acid

metabolite pathway; Panel K, threonine metabolism pathway; Panel L, glycine metabolism pathway; Panel M, summary of regulation of amino acid metabolism pathways. EC numbers are indicated beside genes.

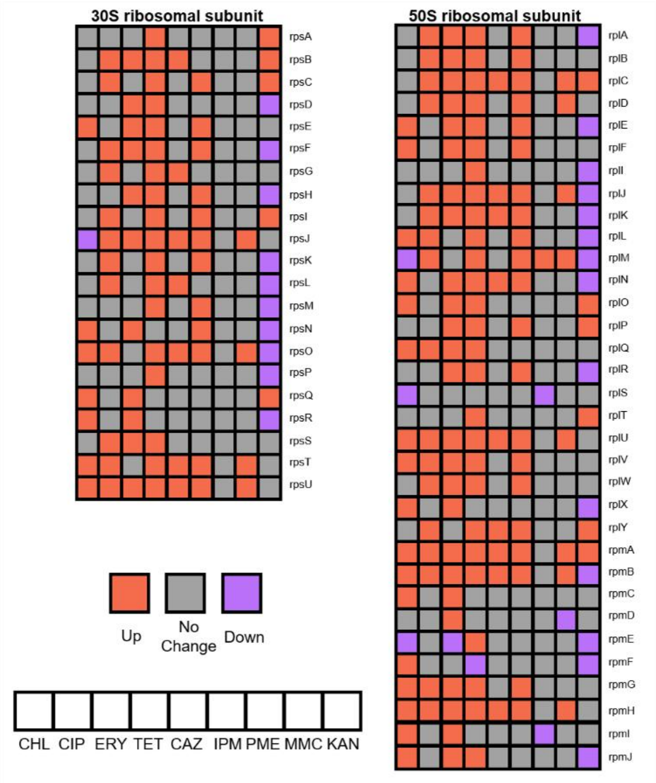

Figure S30. Transcriptomic response of protein biosynthesis.

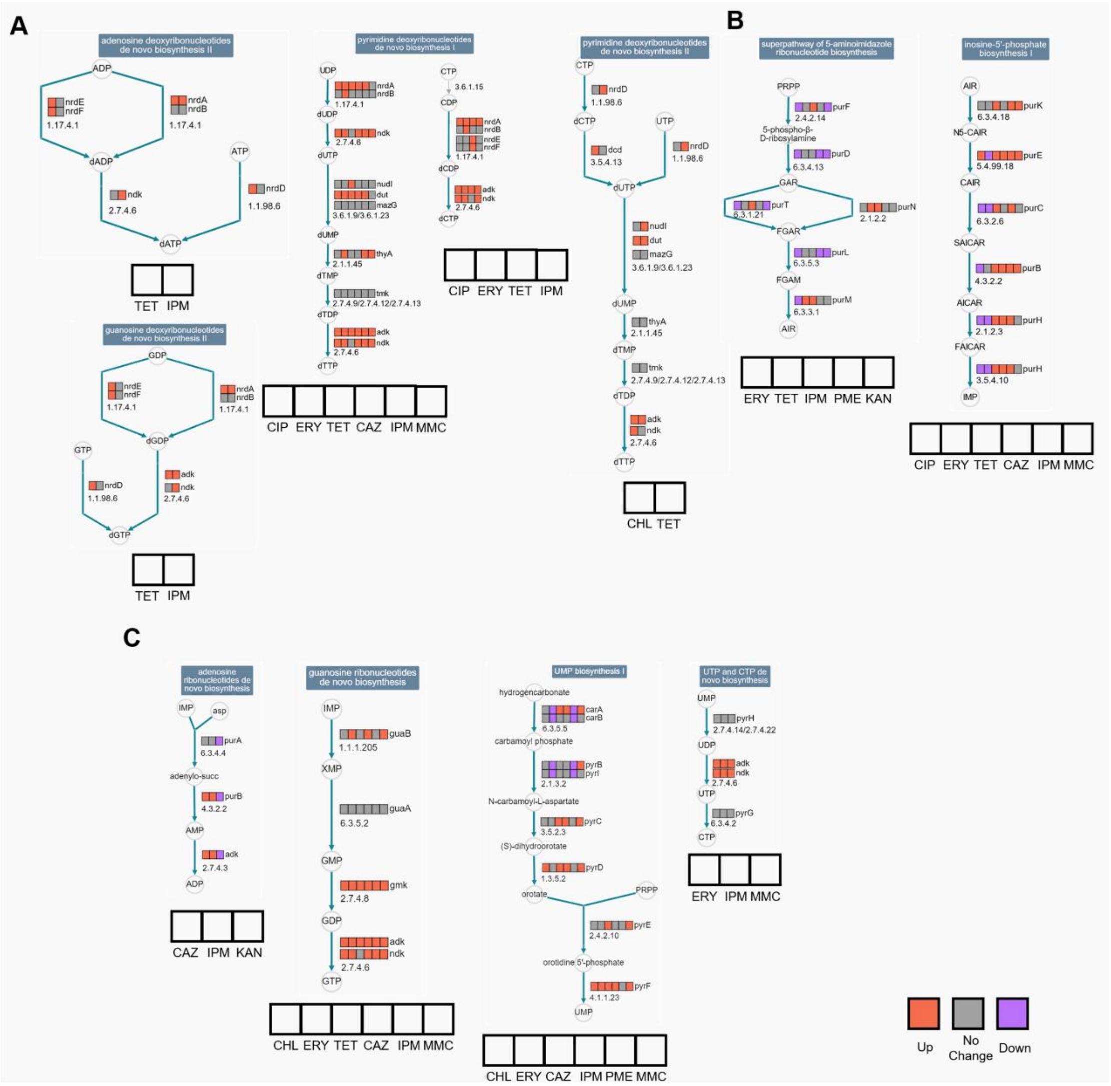

Figure S31. Transcriptomic response of nucleotide biosynthesis pathways. Panel A, dNTP biosynthesis pathways; Panel B, nucleotide precursor biosynthesis pathways; Panel C, NTP biosynthesis pathways. EC numbers are indicated beside genes.

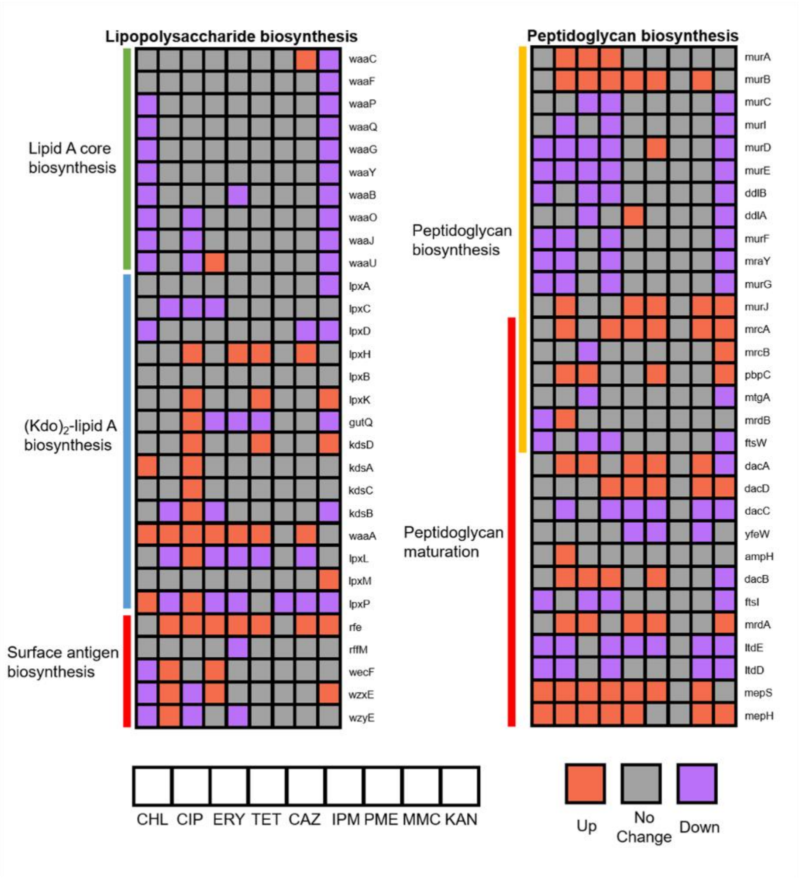

Figure S32. Transcriptomic response of cell wall biosynthesis.

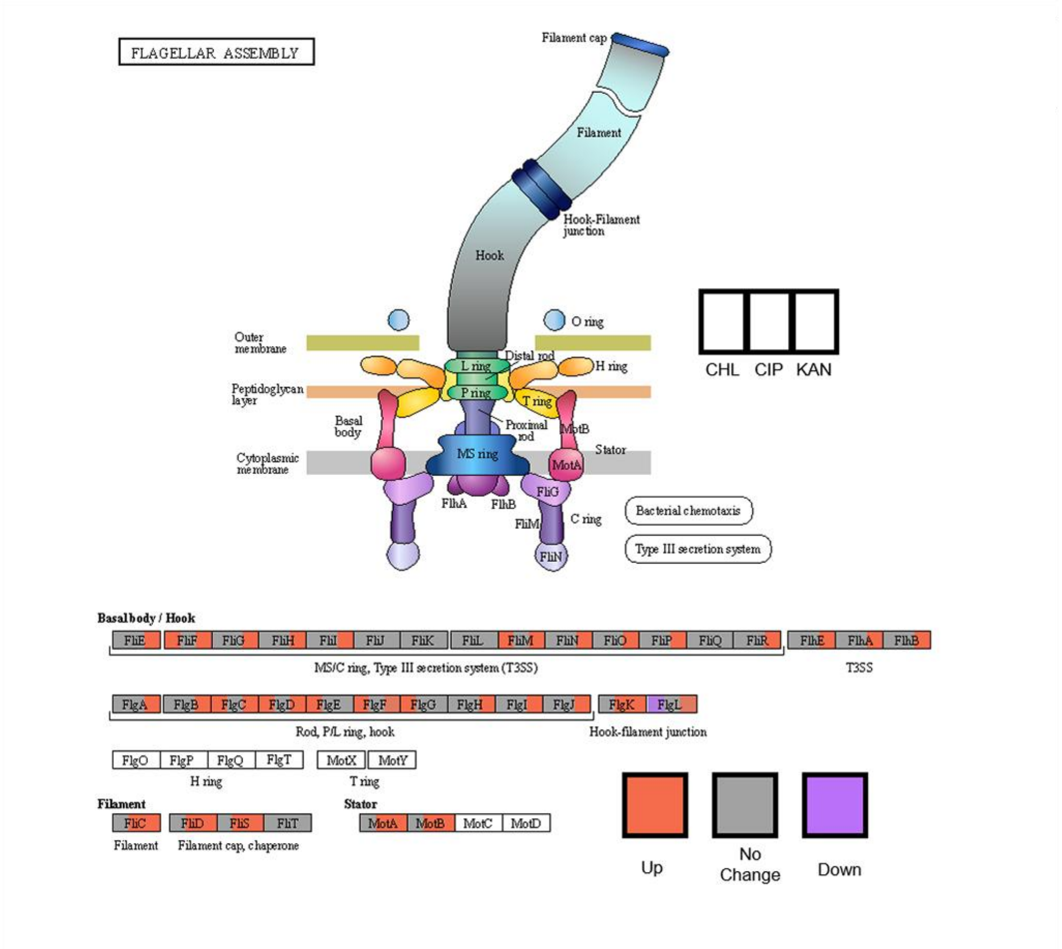

Figure S33. Transcriptomic response of flagella assembly.

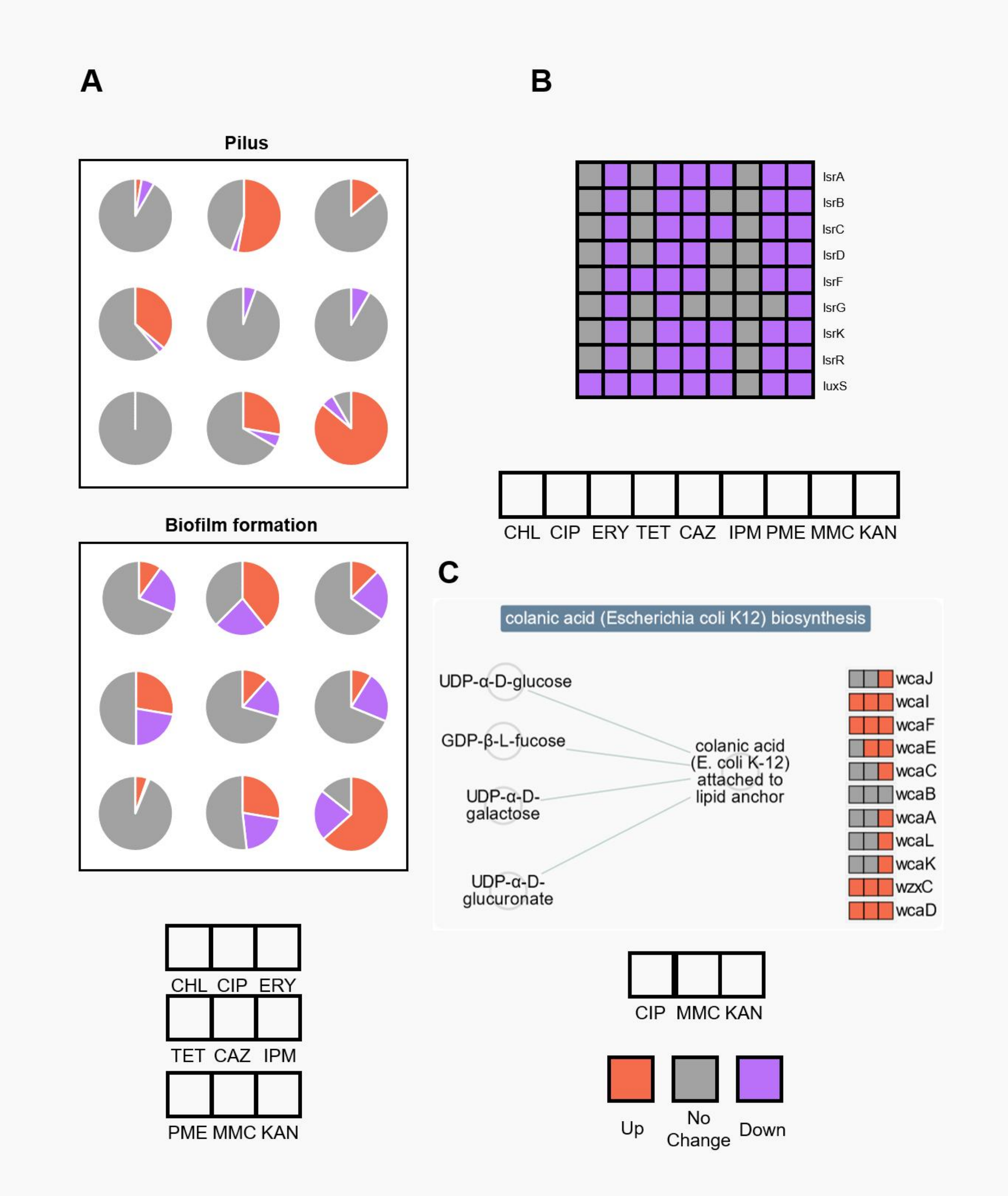

Figure S34. Transcriptomic response of cell-environment communications. Panel A, pilus and biofilm formation; Panel B, quorum sensing; Panel C, colonic acid biosynthesis.
